# Supplementary material for: Space Station conditions are selective but do not alter microbial characteristics relevant to human health
Source: Nat Commun. 2019 Sep 5;10:3990. doi: 10.1038/s41467-019-11682-z (PMC6728350; doi:10.1038/s41467-019-11682-z)
Supplement: Supplementary file 1 — Supplementary Information [file 41467_2019_11682_MOESM1_ESM.pdf]

Supplementary Information

Space Station conditions are selective but do not alter microbial characteristics relevant to human health

Maximilian Mora et al.

Supplementary Fig. 1

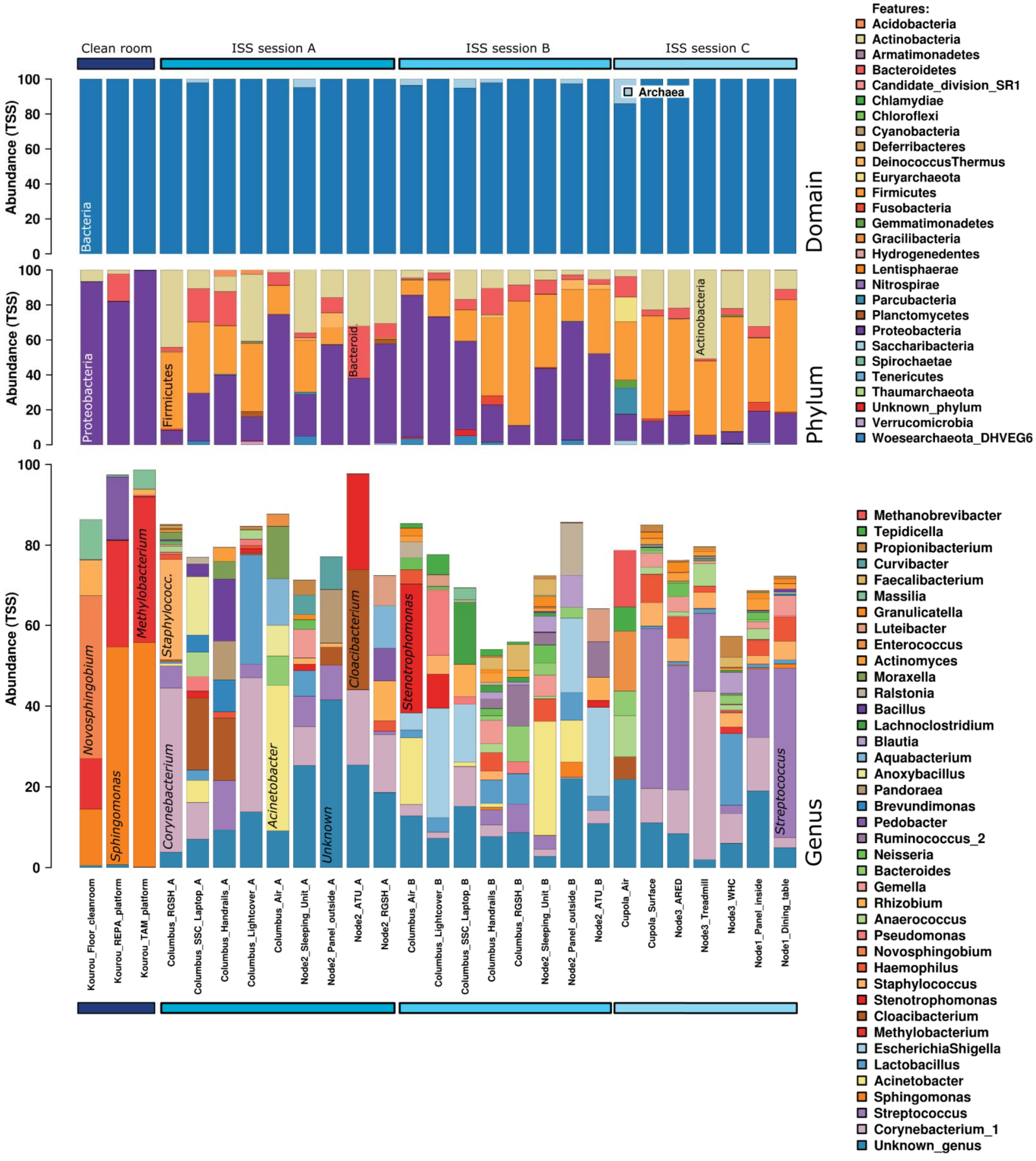

Supplementary Fig. 1: Microbiome composition in cleanrooms (left column) and the ISS sampled during session A, B and C.

Supplementary Fig. 2

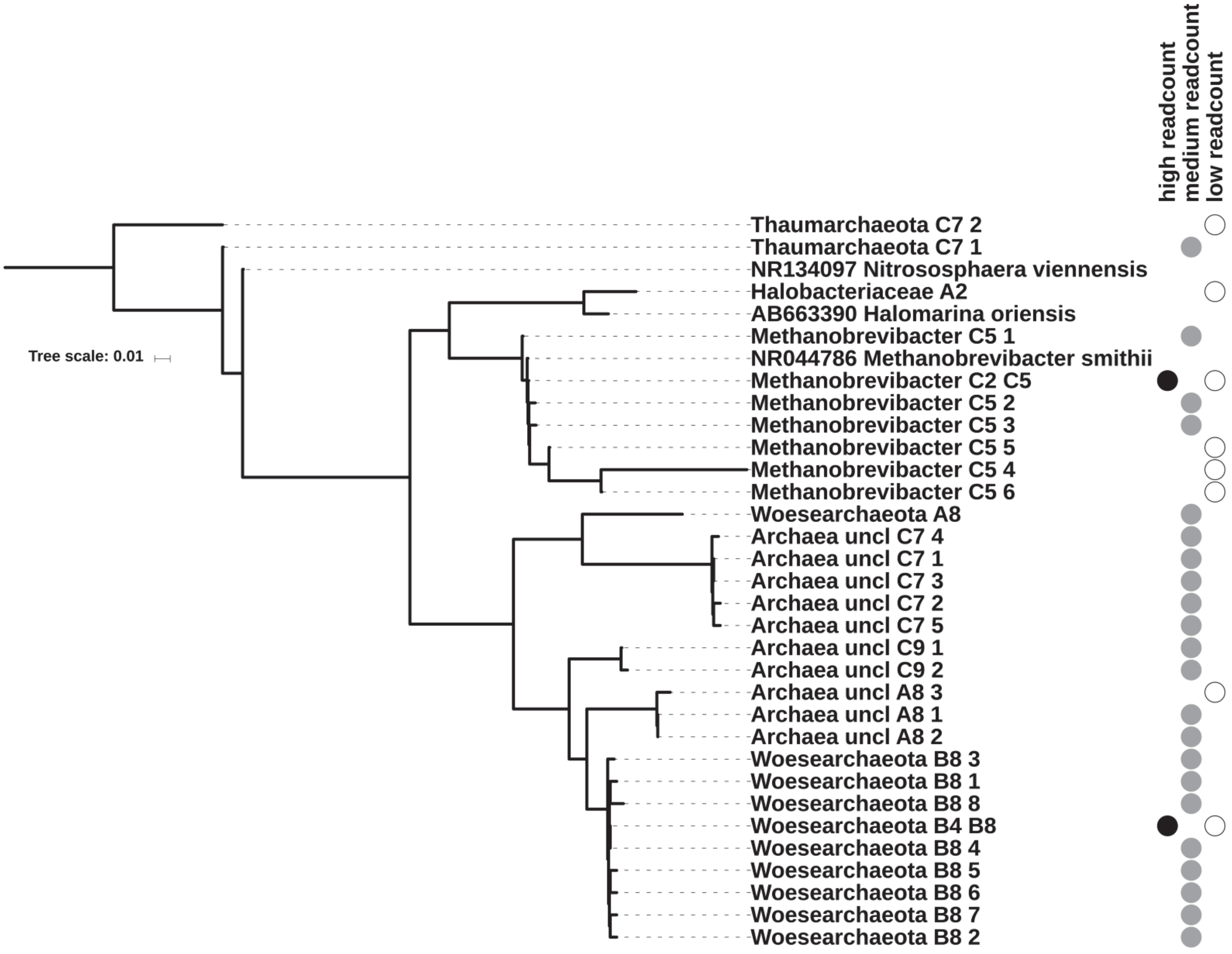

Supplementary Fig. 2: Maximum likelihood tree of archaeal signatures detected by the Archaea-specific 16S rRNA gene sequencing approach: samples were generally dominated by single taxa, therefore read count values are given as a tentative proxy for individual RSV´s relative abundances: **high read count:** >10k reads; **medium read count:** 100-10k reads; **low read counts:** <100 reads**.** AB663390 *Halomarina oriensis*, NR044786 *Methanobrevibacter smithii* and NR134097 *Nitrososphaera viennenis* represent sequences of type strains of closest cultivated neighbours.

Supplementary Fig. 3

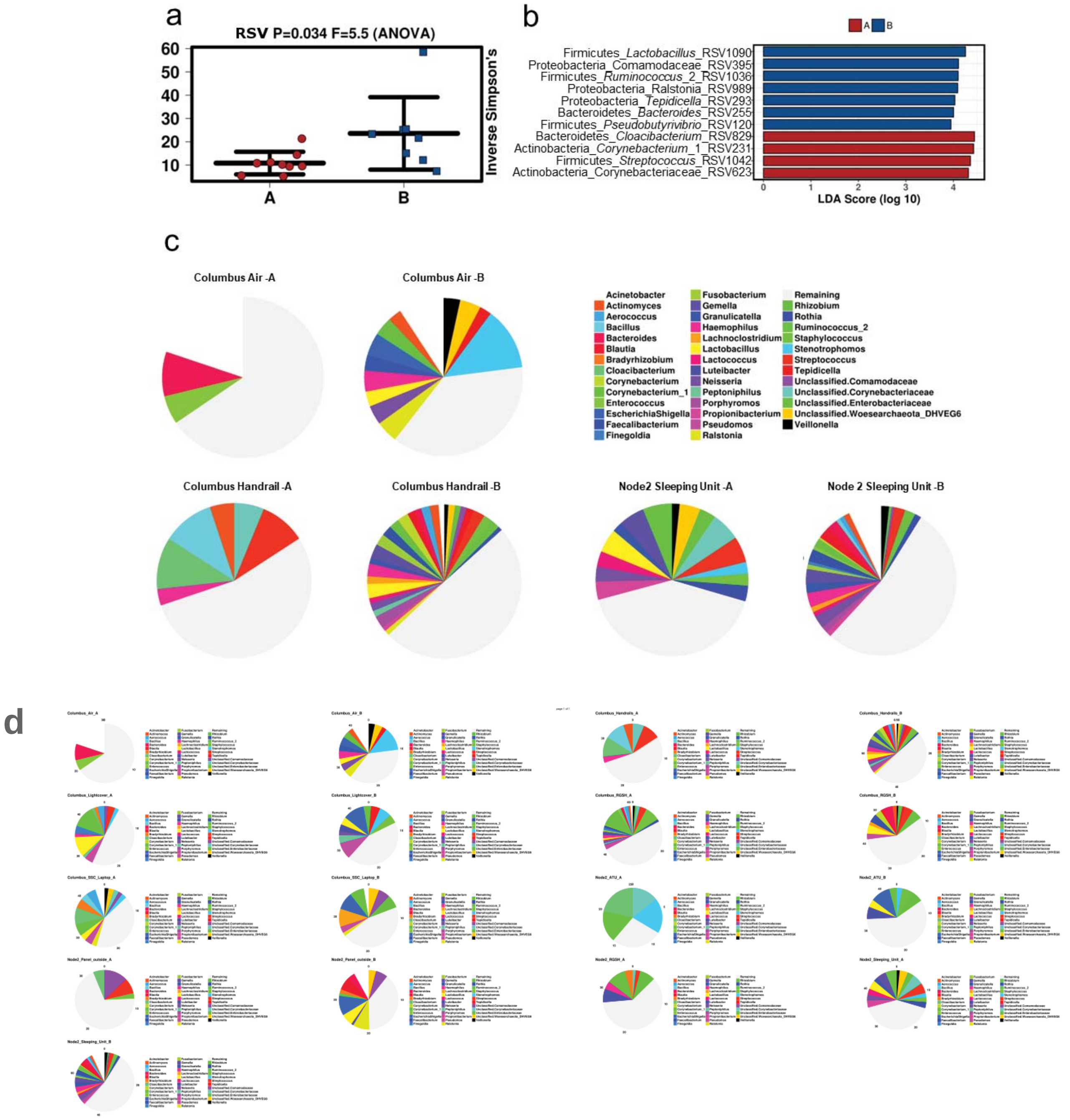

Supplementary Fig. 3: Temporal transitions in microbiome taxonomic diversity (same locations were sampled, universal approach). a) Inverse Simpson's index, indicating a significantly different microbiome diversity in samples from session A and B. Error bars reflect standard deviation. b) LEfSe analysis (300 most abundant taxa), comparing session A and B. c) Selected pie charts (close-up) created including the top 40 most abundant microbial taxa for selected samples. "Columbus Air -A" refers to sample taken from Columbus module: air, in session A. d) All pie charts, created including the top 40 most abundant microbial taxa, for all sampled areas from session A and B.

Supplementary Fig. 4

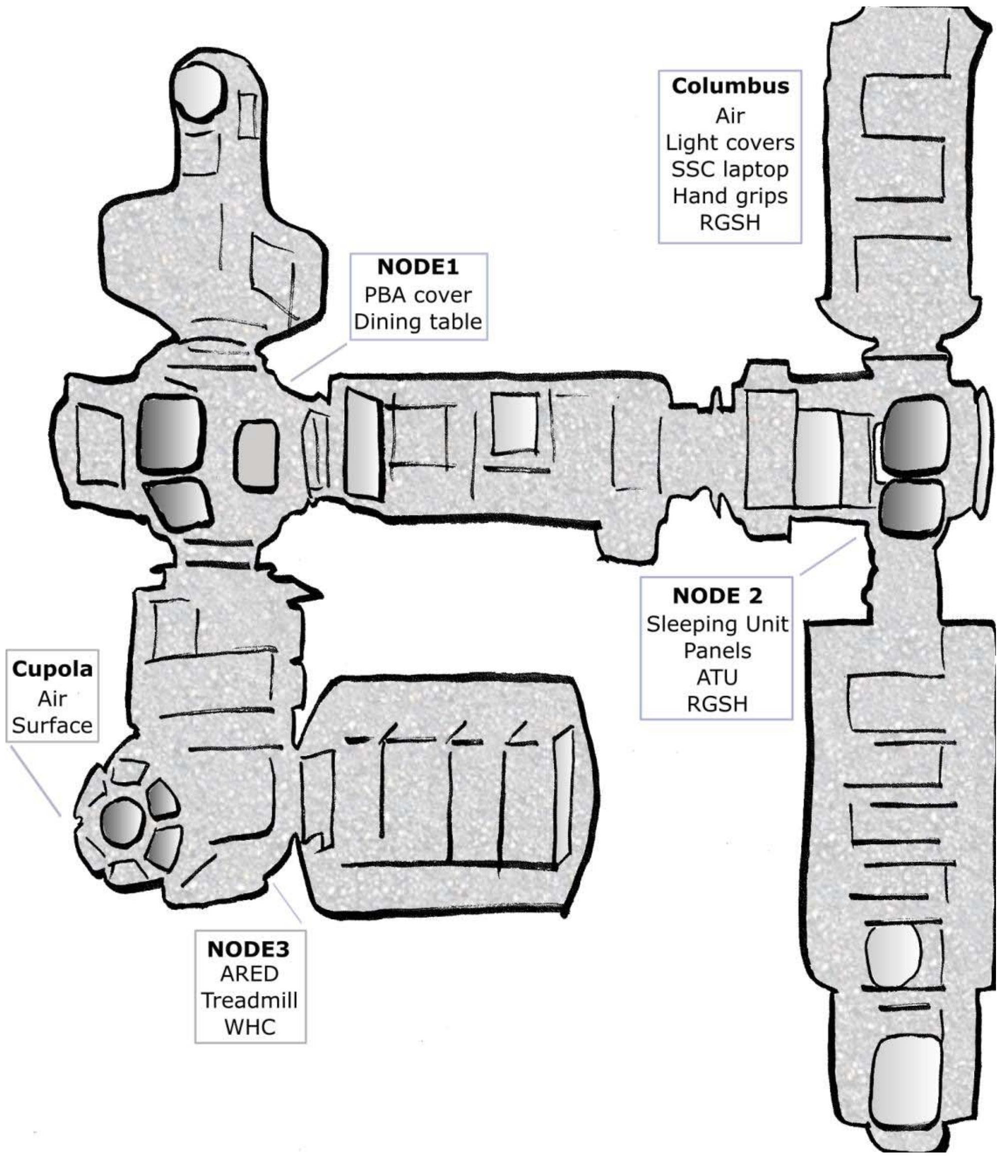

Supplementary Fig. 4: Schematic drawing of NASA, ESA and JAXA modules of the ISS, with sampling locations (modules and detailed locations).

Supplementary Fig. 5

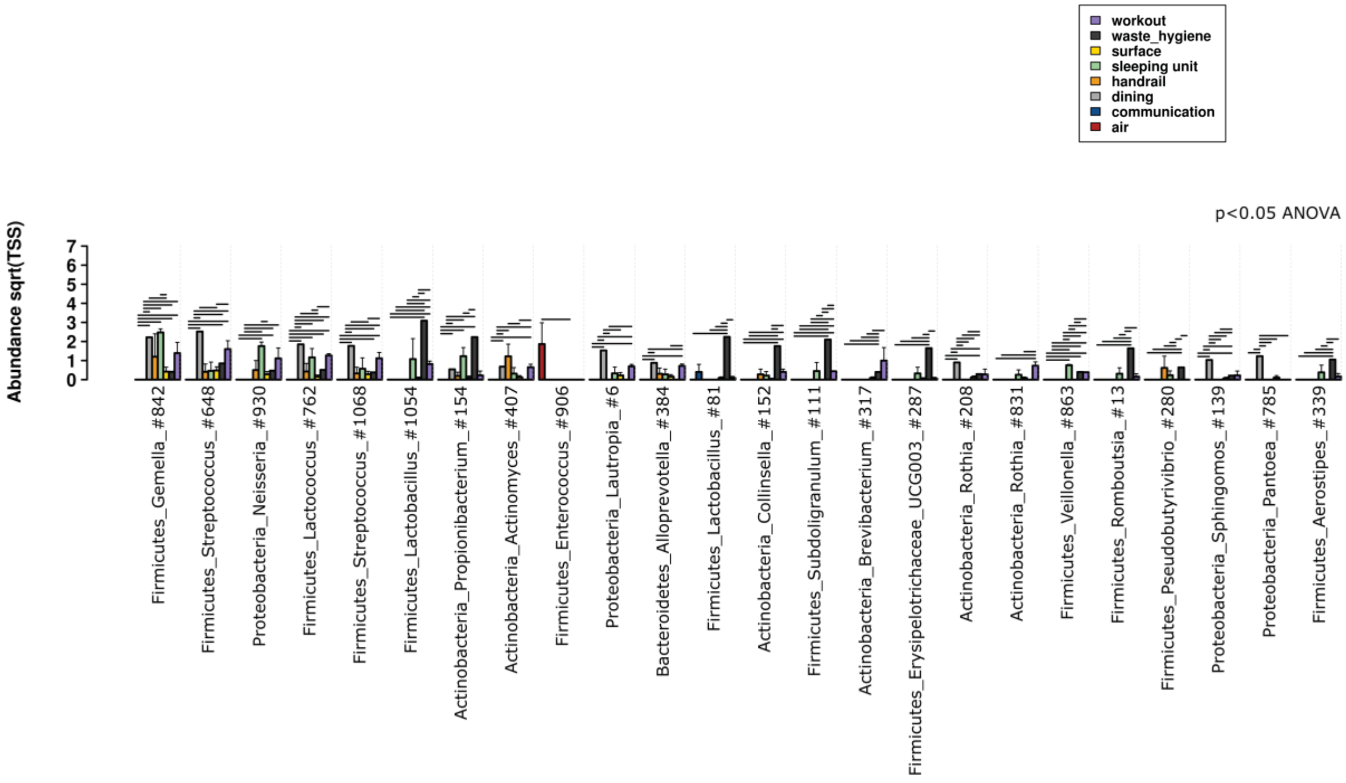

Supplementary Fig. 5: ANOVA plot analysis of different types of locations within the ISS and specifically associated RSVs. Error bars reflect standard deviation.

Supplementary Fig. 6

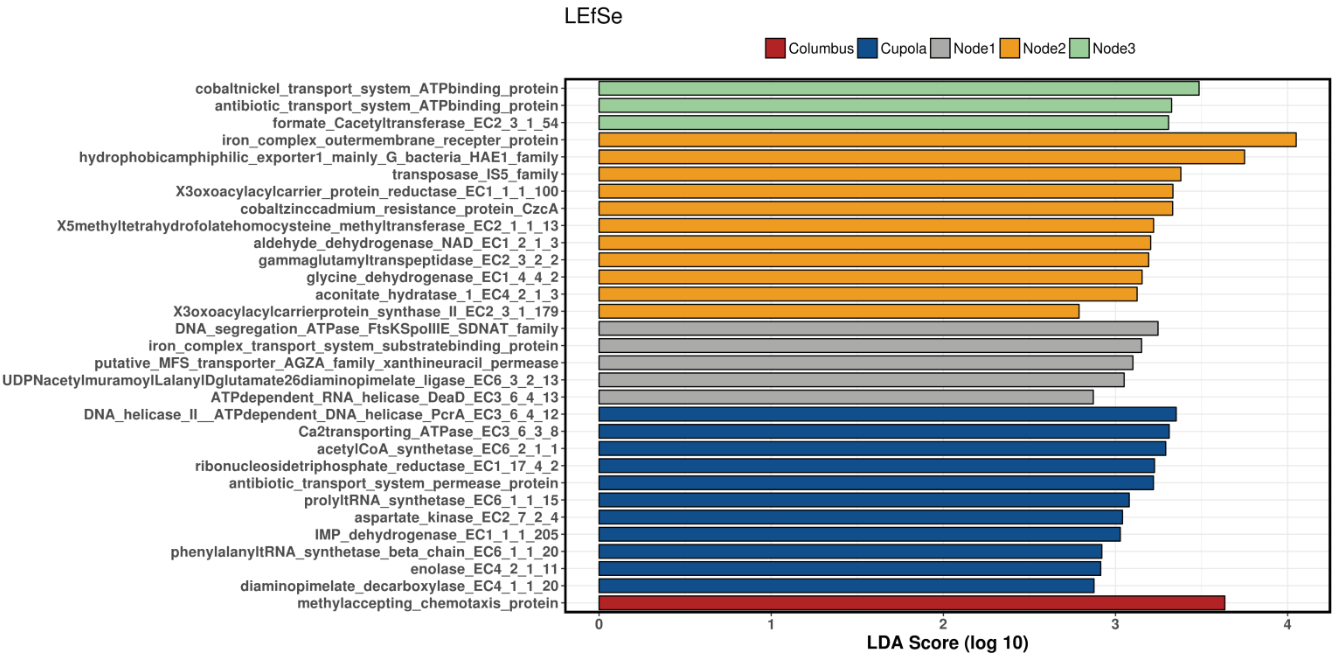

Supplementary Fig. 6: LEfSe analysis of predicted functions (Tax4fun), grouped by sampling location (module).

Supplementary Fig. 7

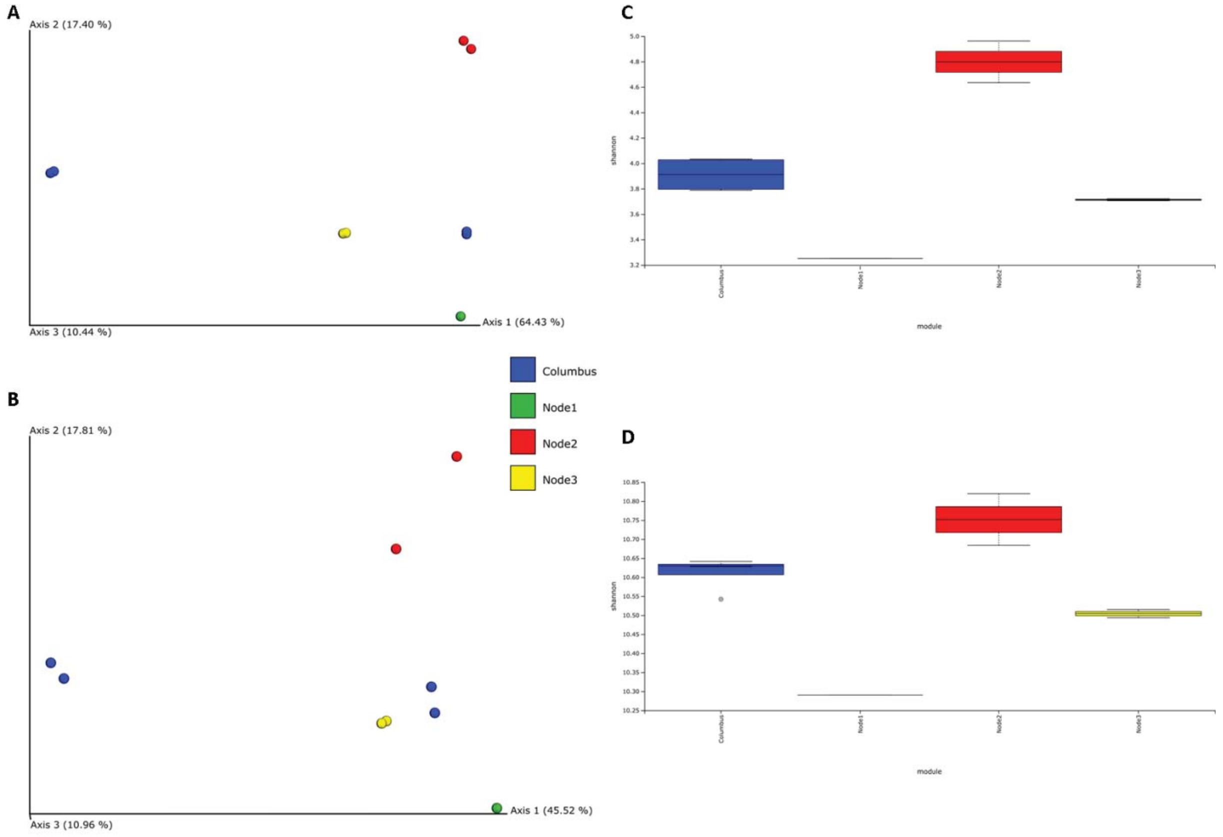

Supplementary Fig. S7: Alpha and Beta diversity estimates of the read-centric shotgun metagenomic dataset. A) PCoA based on Bray-Curtis distances of assigned taxa. B) PCoA based on Bray-Curtis distances of assigned functions. C) Shannon alpha diversity estimates of assigned taxa. D) Shannon alpha diversity estimates of assigned functions. For all panels: blue (Columbus), green (NODE1), red (NODE2), yellow (NODE3).

Supplementary Fig. 8

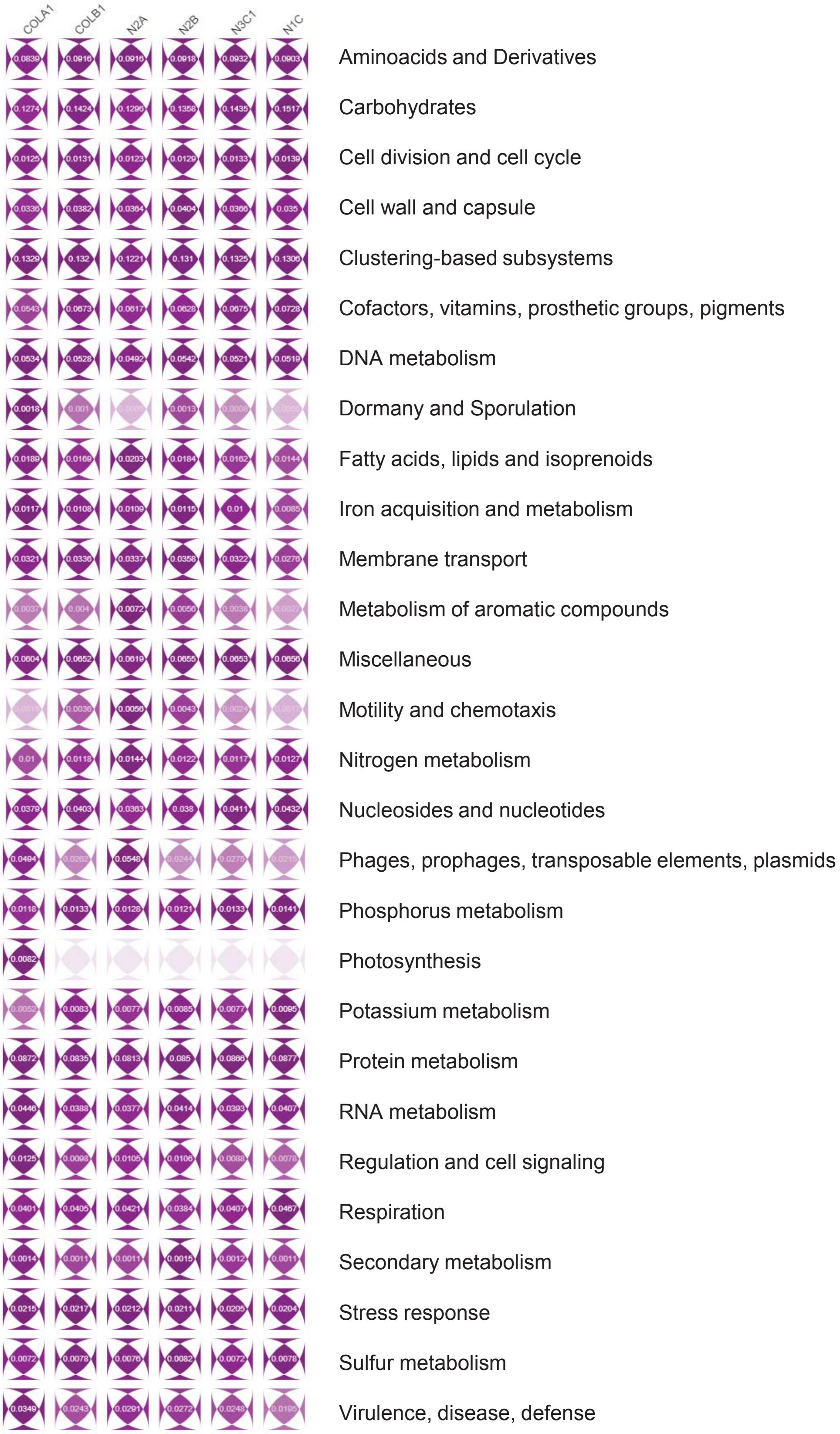

Supplementary Fig. 8: Distribution of microbial functions inferred from metagenomic information. COLA1: Columbus Session A. COLB1: Columbus Session B. N2A: Node 2 Session A. N2B: Node 2 Session B. N3C1: Node 3 Session C. N1C: Node 1 Session C.

Supplementary Fig. 9

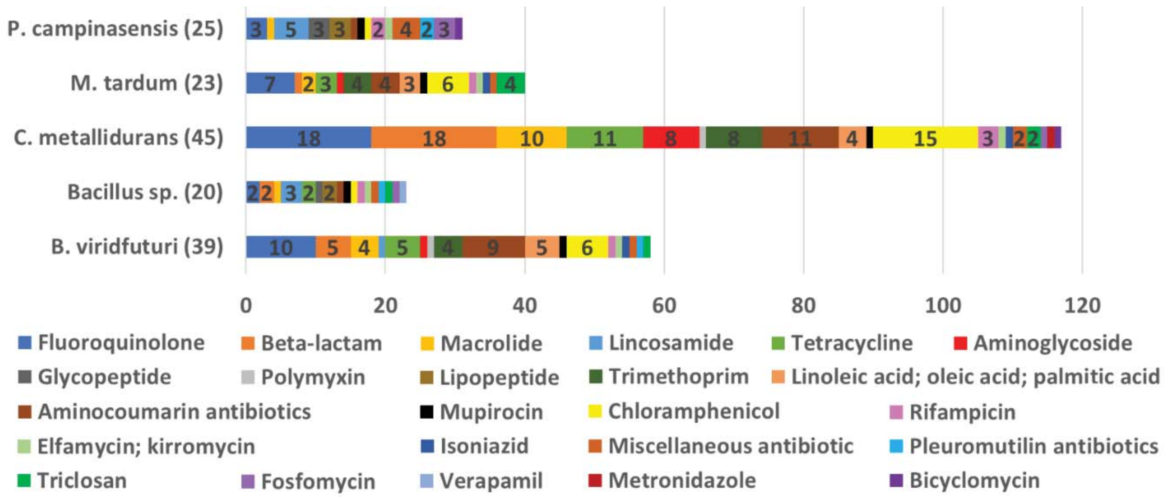

Supplementary Fig. 9: Summary of the antibiotic resistance genes (ARG) detected in the sequenced genomes. The number (in brackets) is the total number of detected ARG in the respective genome and the colored columns show the groups of antibiotics against which the detected ARG are known to provide resistance. Numbers within a colored column indicate how many of the detected ARG provide resistance against this type of antibiotic; in case of one ARG was found, no number is given. As some ARG, especially the multidrug efflux pumps which were found in a high number in *C. metallidurans* and *B. viridfuturi*, may be able to provide resistance against multiple kinds of antibiotics, the sum of the numbers within the colored columns may exceed the total number of detected antibiotic resistance genes per organism. The number of ARG as well as of inferred antibiotic resistances was the same in the sequenced *Bacillus* genomes, which is why they are summarized as one *Bacillus sp.* in this graph.

Supplementary Fig. 10

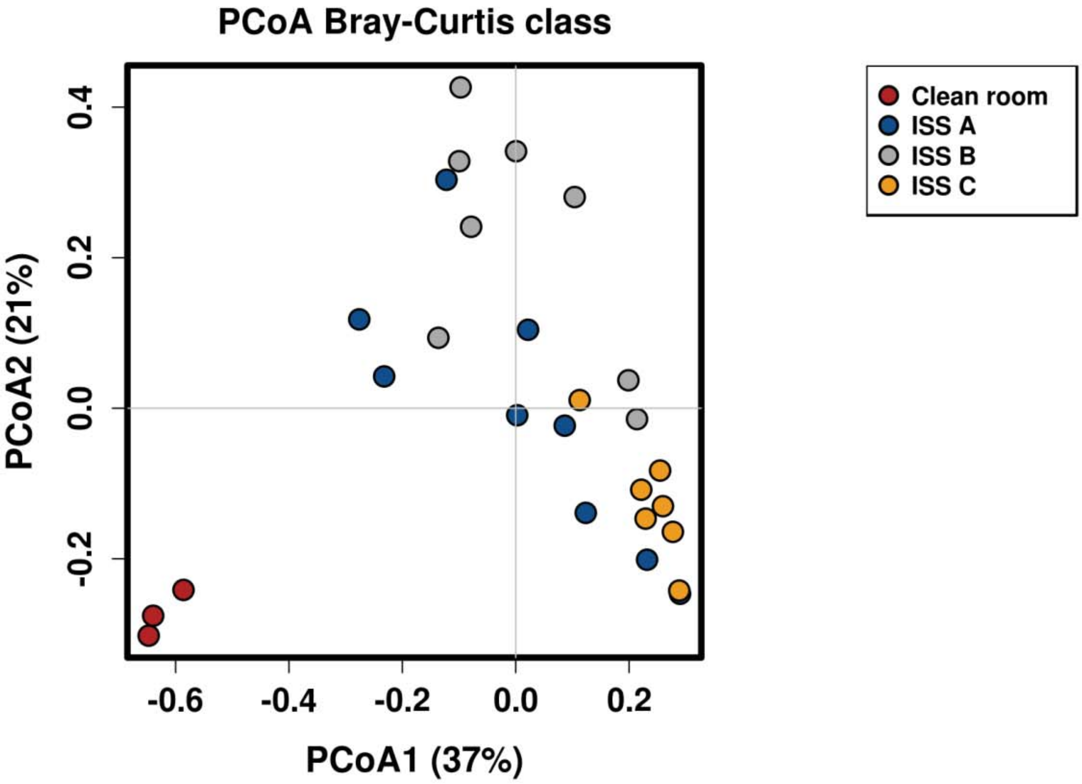

Supplementary Fig. 10: PCoA plot of microbiome compositions of clean room and ISS samples

Supplementary Fig. 11

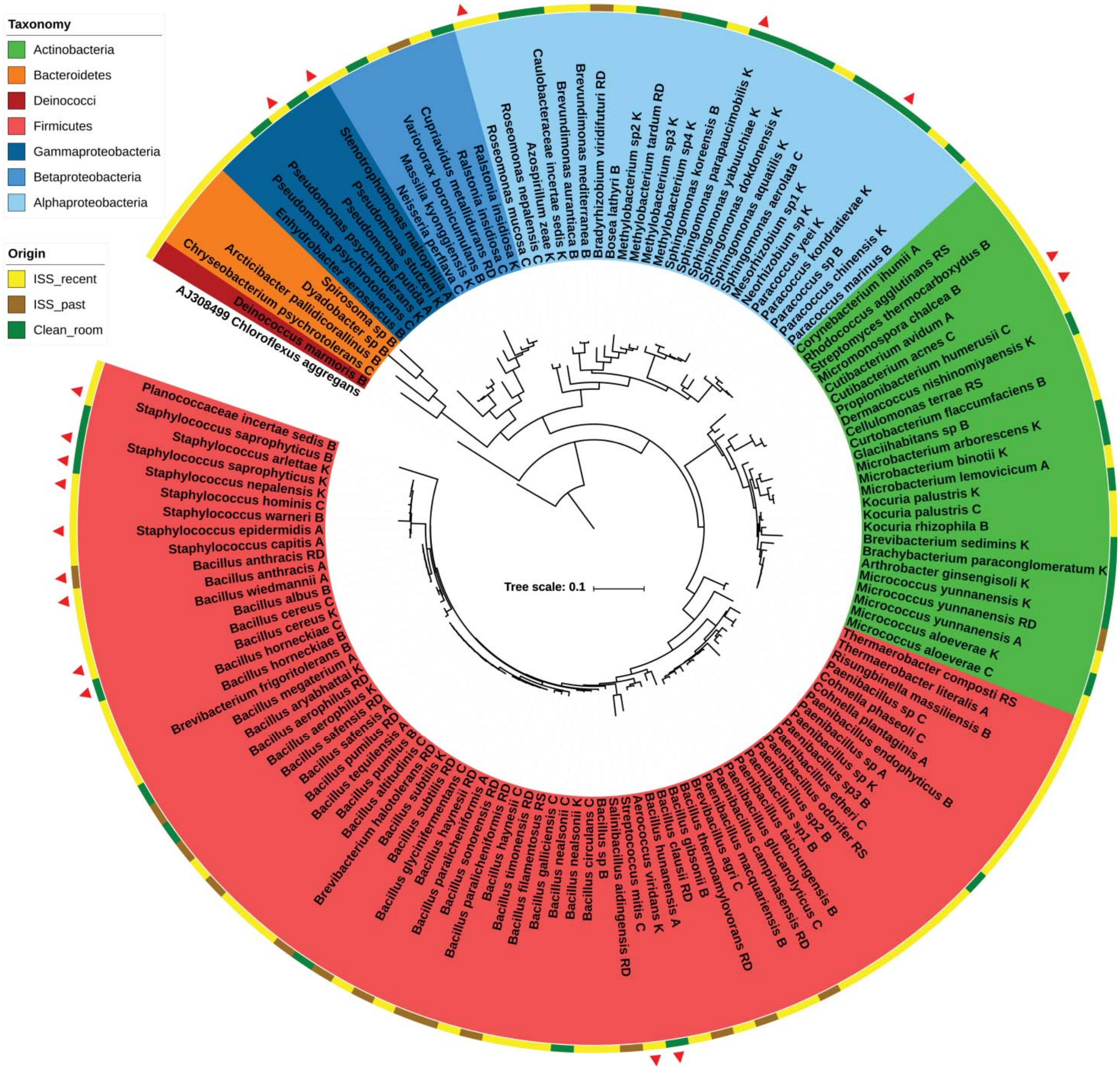

Supplementary Fig. 11: Phylogenetic tree of ISS isolates from this ("ISS_recent") and our previous study ("ISS_past"), including clean room isolates obtained during the Kourou/ ATV sampling campaign (this study; "Clean_room"). Red triangles indicate a biosafety risk classification of S2. Isolates which are not assigned a species had less than 98.5% 16S rRNA gene sequence similarity to their closest described neighbour.

Supplementary Fig. 12

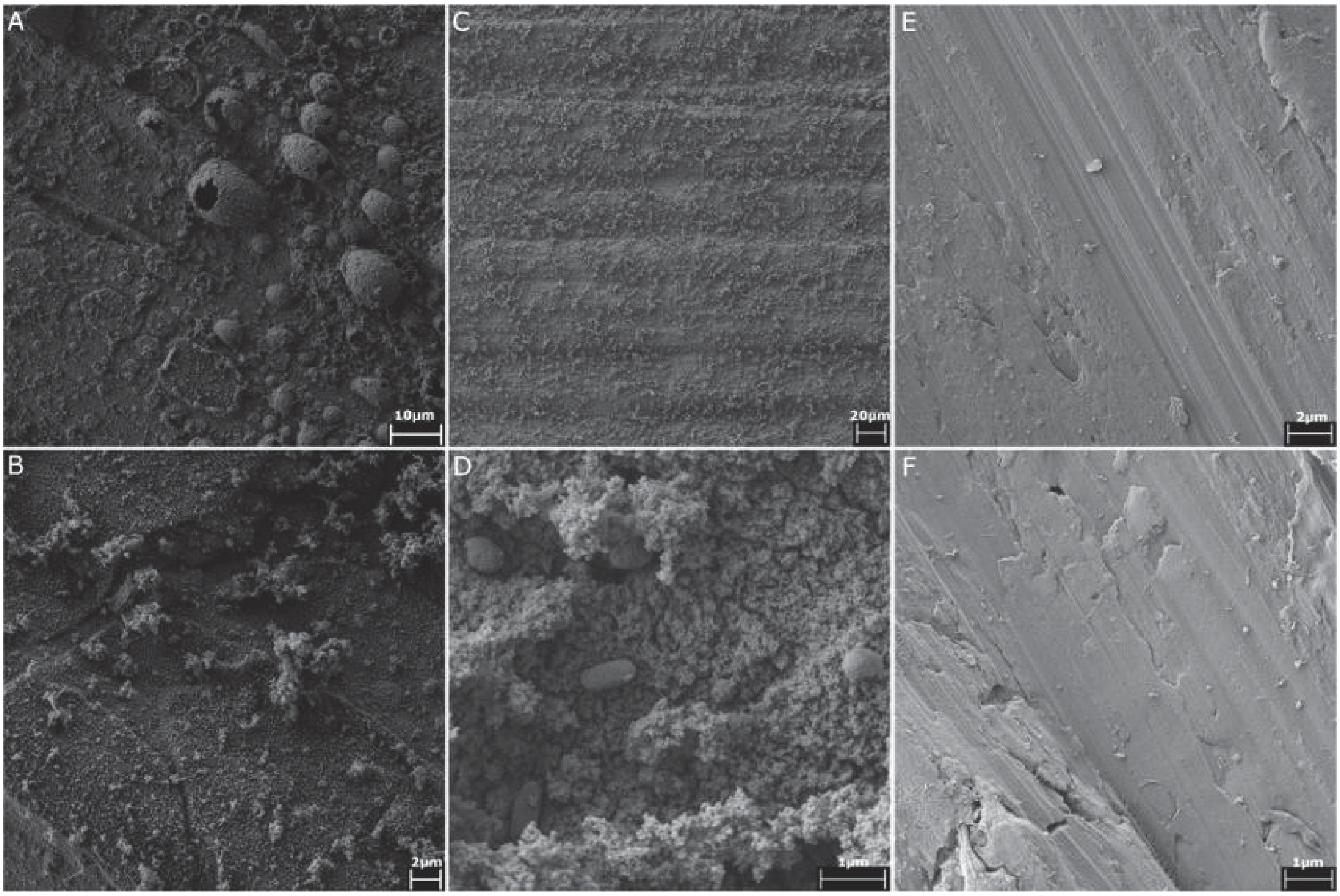

Supplementary Fig. 12: **Scanning electron micrographs of polished and untreated aluminium copper magnesium alloy EN AW 2219 which was co-incubated for six weeks with bacteria isolated from the ISS: A-B:** Coincubation with *Bacillus paralicheniformis*; **C-D:** Coincubation with *Cupriavidus metallidurans*; **E-F:** Negative control of the same alloy kept in sterile medium for six weeks**.**

Supplementary Fig. 13

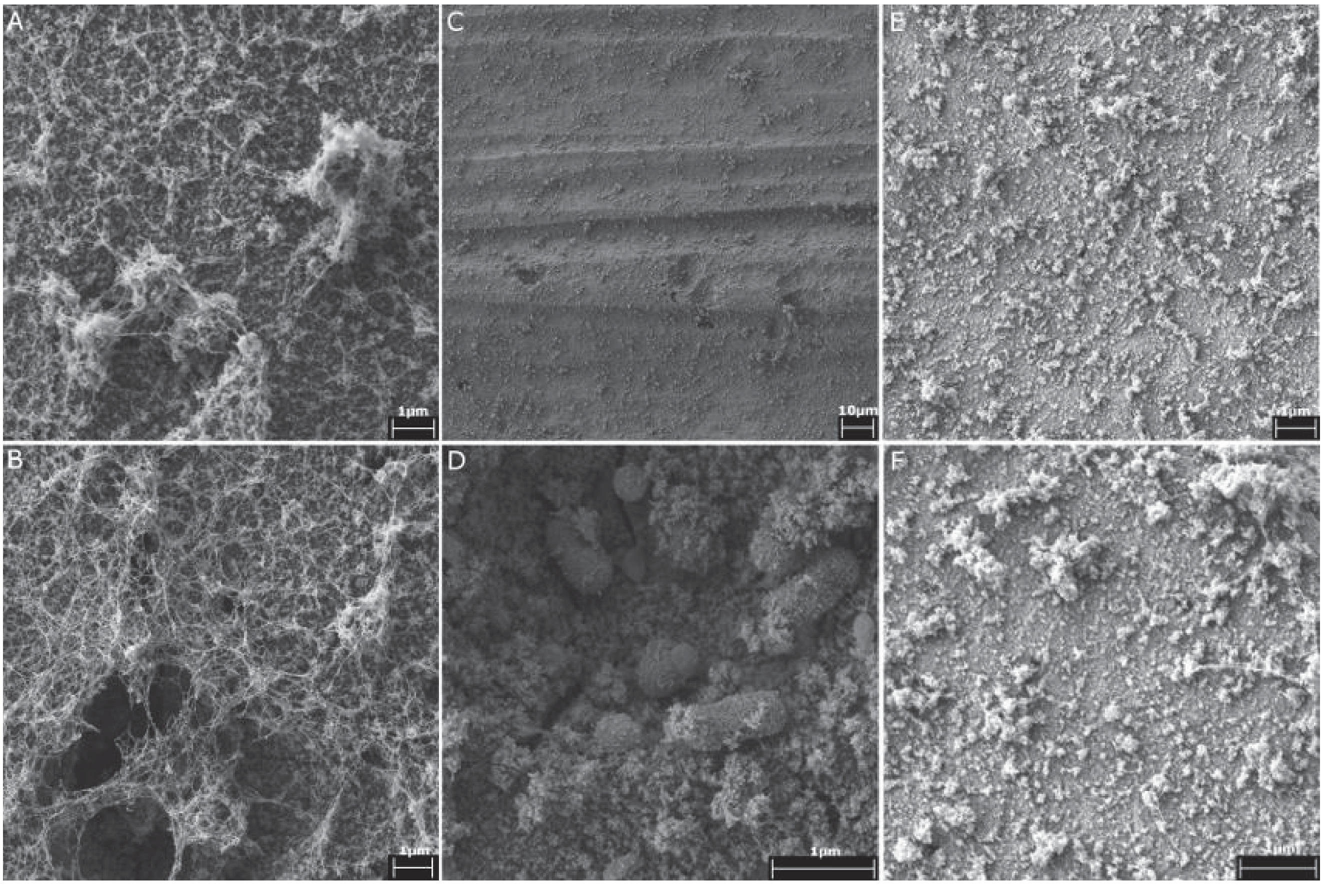

Supplementary Fig. 13: **Scanning electron micrographs of polished and anodized aluminium copper magnesium alloy EN AW 2219 which was co-incubated for six weeks with bacteria isolated from the ISS: A-B:** Coincubation with *Bacillus paralicheniformis*; **C-D:** Coincubation with *Cupriavidus metallidurans*; **E-F:** Negative control of the same alloy kept in sterile medium for six weeks**.**

Supplementary Fig. 14

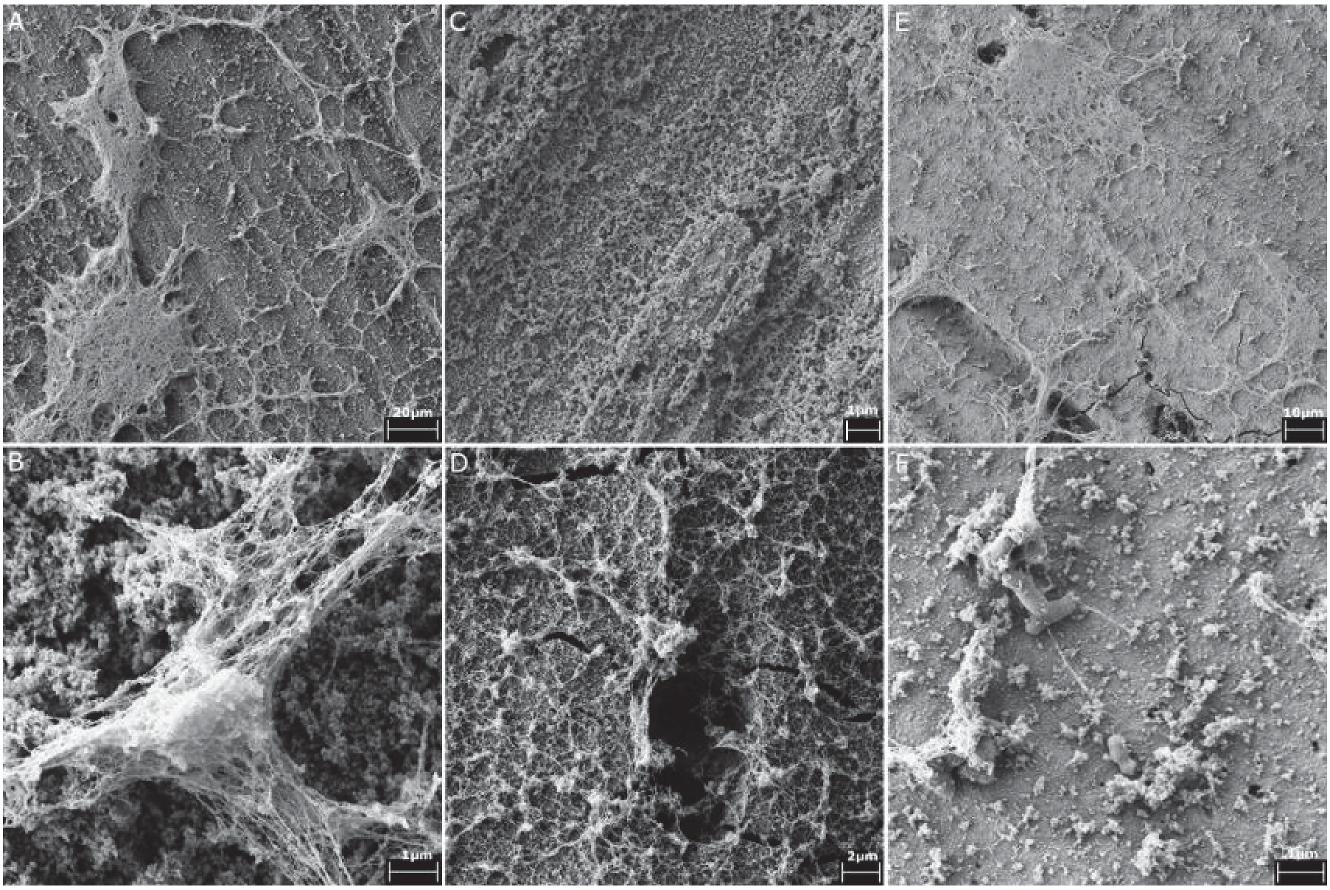

Supplementary Fig. 14: **Scanning electron micrographs of polished untreated and of anodized aluminium copper magnesium alloy EN AW 2219 which was anaerobically co-incubated for six weeks with bacteria isolated from the ISS: A-B:** Untreated alloy with *Cutibacterium avidum*; **C:** Negative control of untreated alloy kept in sterile medium for six weeks; **D;** Negative control of anodized alloy kept in sterile medium for six weeks; **E-F:** Anodized alloy with *Cutibacterium avidum*.

Supplementary Fig. 15

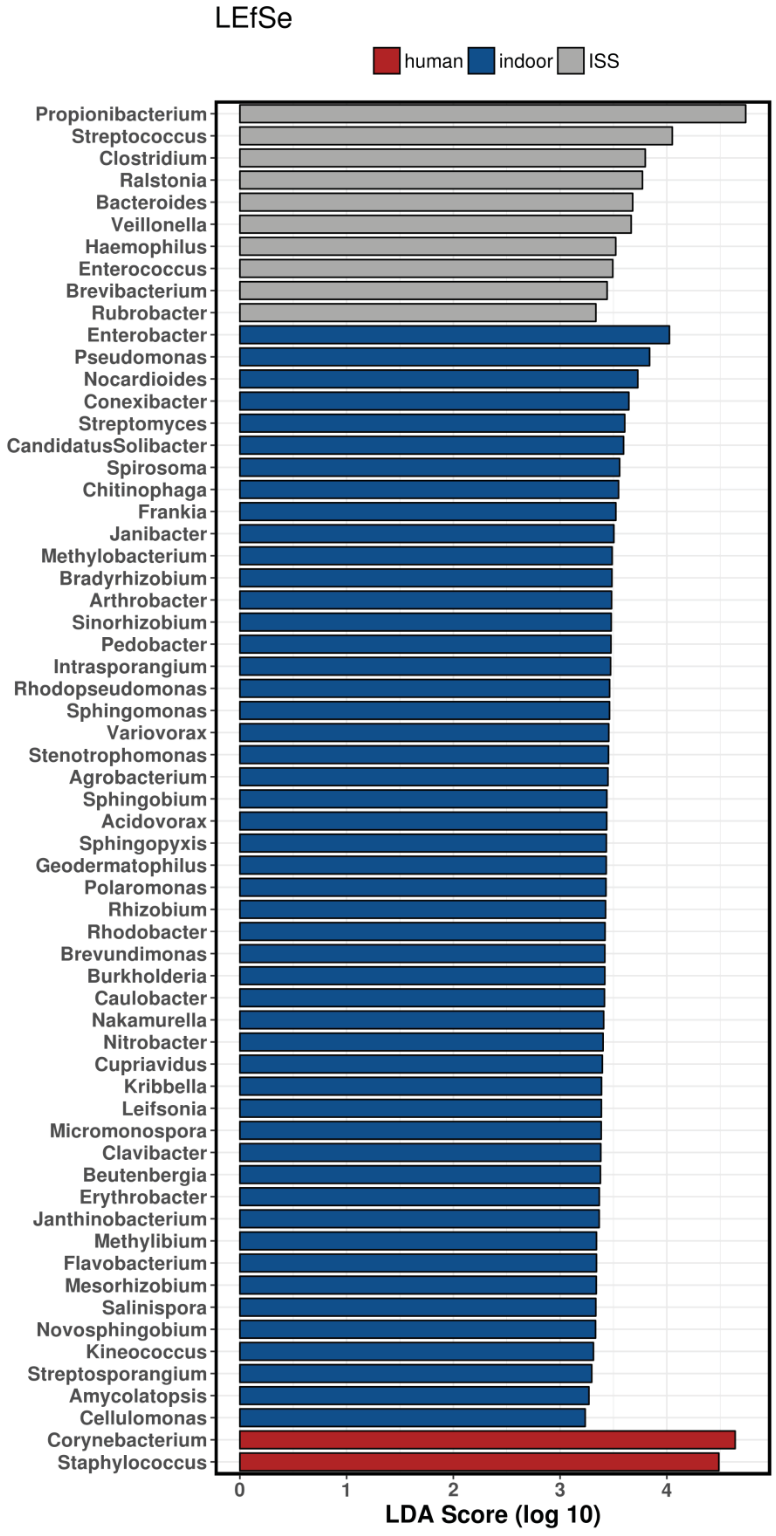

Supplementary Fig. 15: **LEfSE analysis** including datasets derived from Lax *et al.,* indentifying biomarker signatures for terrestrial indoor environments, human surfaces or ISS samples.

Supplementary Fig. 16-20 are described in more detail in Note 2.

Supplementary Fig. 16

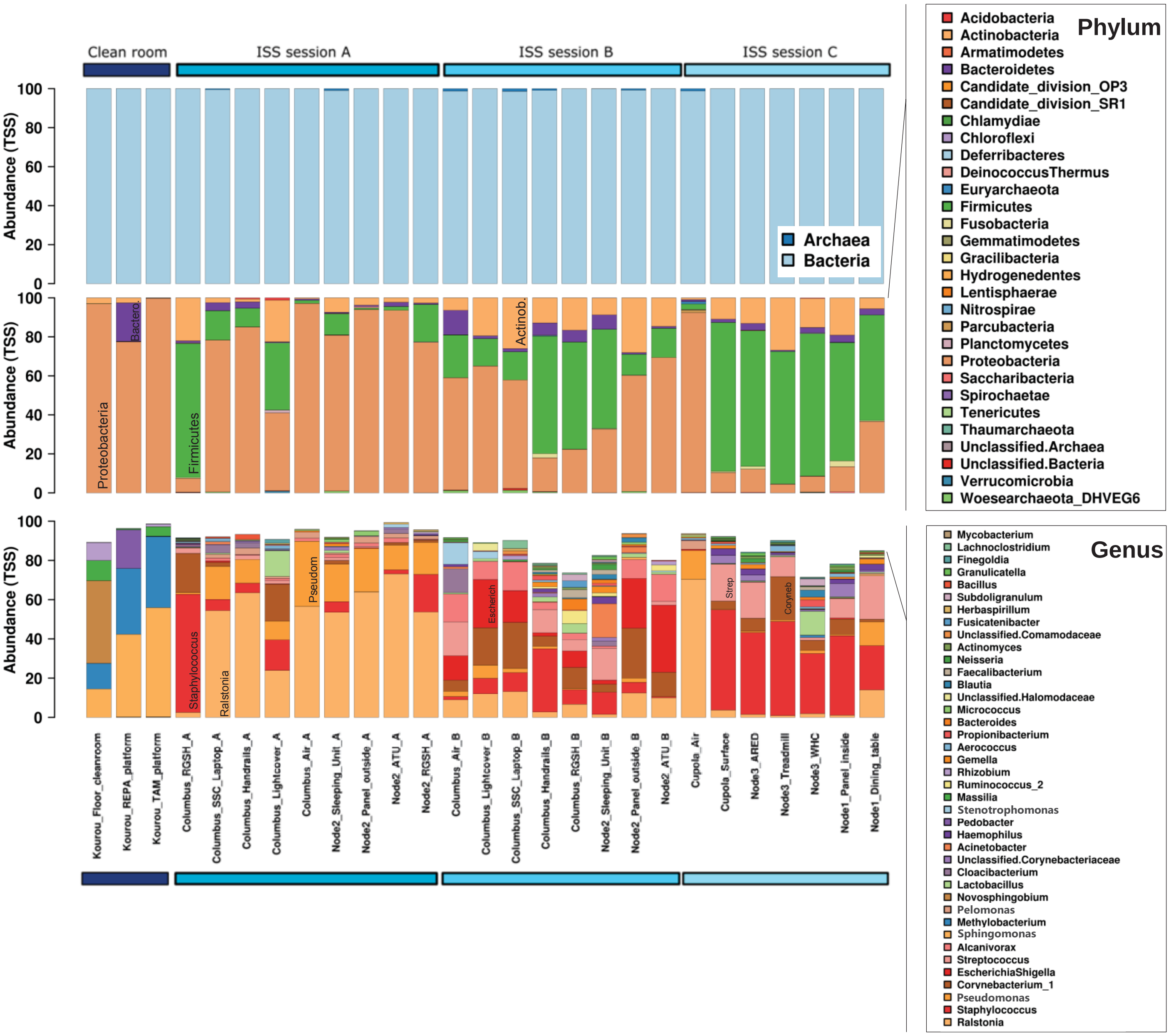

Supplementary Fig. 16 (refers to Fig.1, main manuscript): Microbiome composition in cleanrooms (left column) and the ISS sampled during session A, B and C (decontam- dataset).

Supplementary Fig. 17

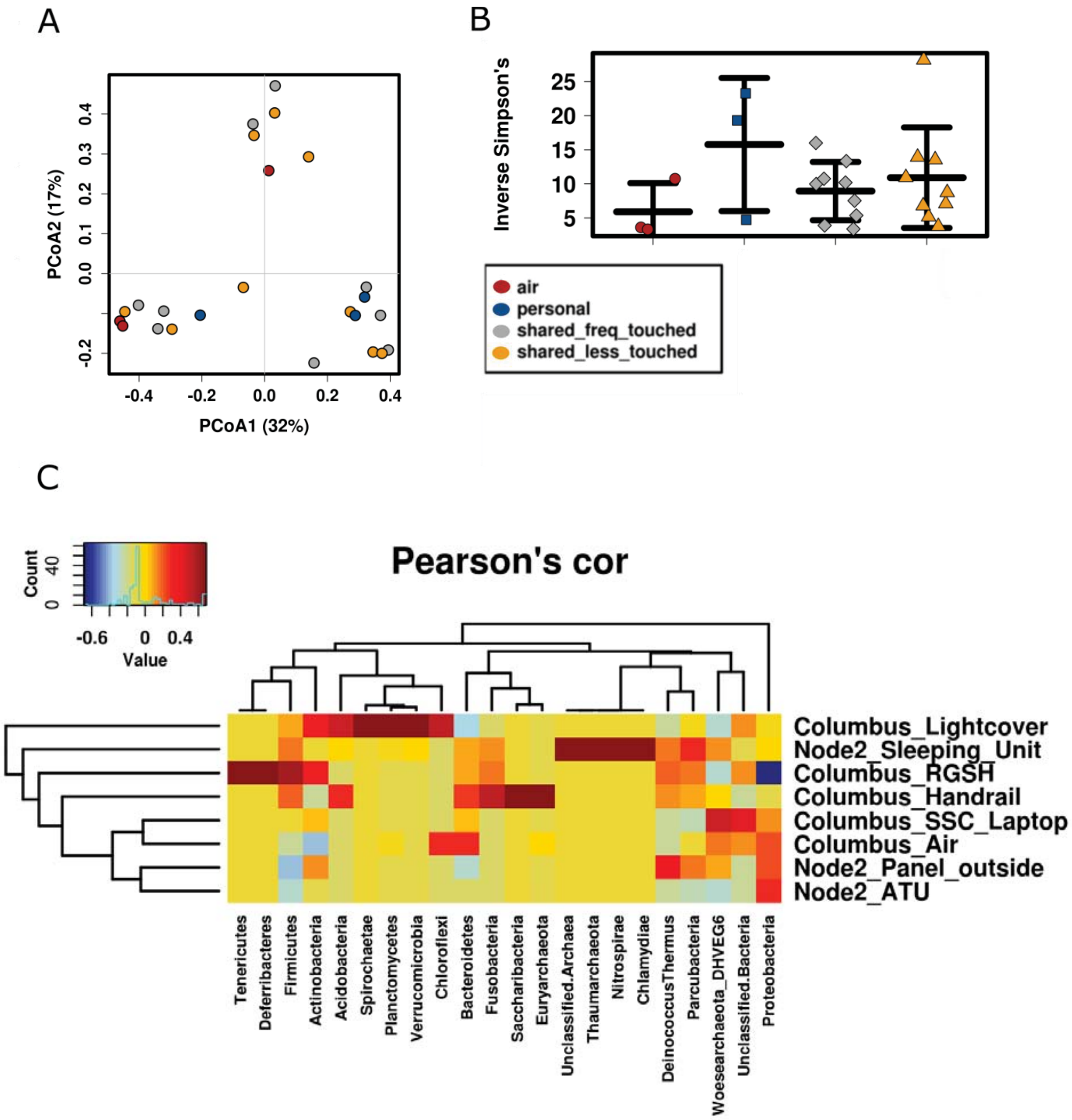

Supplementary Fig. 17 (refers to Fig. 3, main manuscript): Microbiome composition according to sample categories and sample types. A) PCoA plot (based on Bray-Curtis) indicating a separate grouping of personal microbiomes. B) The highest diversity of microbial signatures was observed in microbiome samples from personal areas. The lowest diversity was detected in air samples (sample depth rarefied to 9092 reads). Error bars reflect standard deviation. C) Hierarchical cluster analysis (Pearson's correlation; universal microbiome data set). Certain microbial phyla were found to correlate with specific sampling sites.

Supplementary Fig. 18

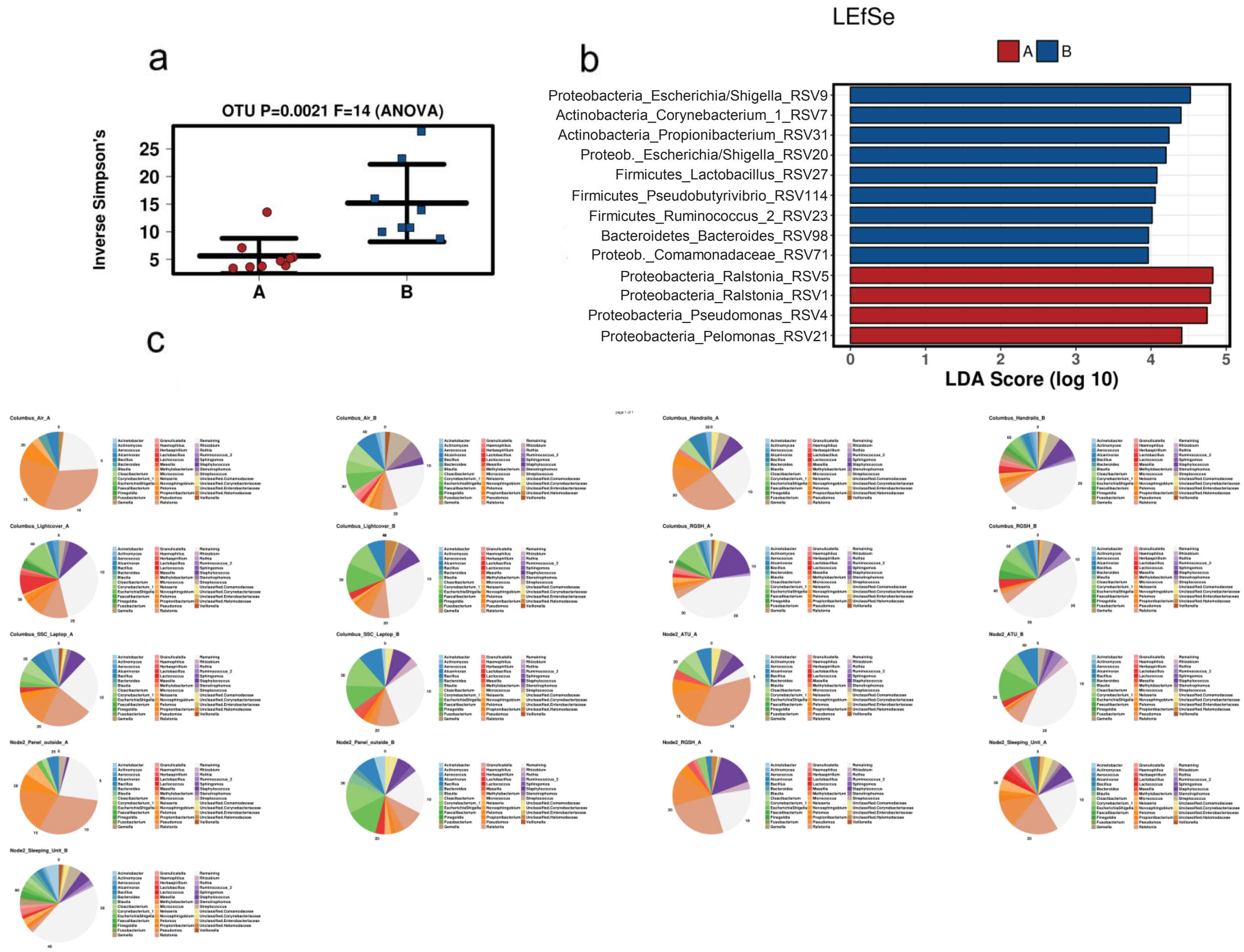

Supplementary Fig. 18 (refers to Supplementary Fig. 3, main manuscript): Temporal transitions in microbiome taxonomic diversity (same locations were sampled, universal approach). a) Inverse Simpson's index, indicating a significantly different microbiome diversity in samples from session A and B. Error bars reflect standard deviation. b) LEfSe analysis (300 most abundant taxa), comparing session A and B. c) Pie charts created including the top 40 most abundant microbial taxa for selected samples. "Columbus Air – A" refers to sample taken from Columbus module: air, in session A.

Also in this dataset, the diversity increased significantly (p=0.0021), along with a significantly increased evenness value (p=0.000056). ANOSIM analysis confirmed a significantly different composition of the samples taken in session A and B (p=0.001). LEfSe analysis (targeting the 300 most abundant genera) confirmed a substantial increase in signatures belonging to typically gastrointestinal tract-associated genera *Escherichia/Shigella*, *Ruminococcus_2* and *Pseudobutyrivibrio* towards session B.

Supplementary Fig. 19

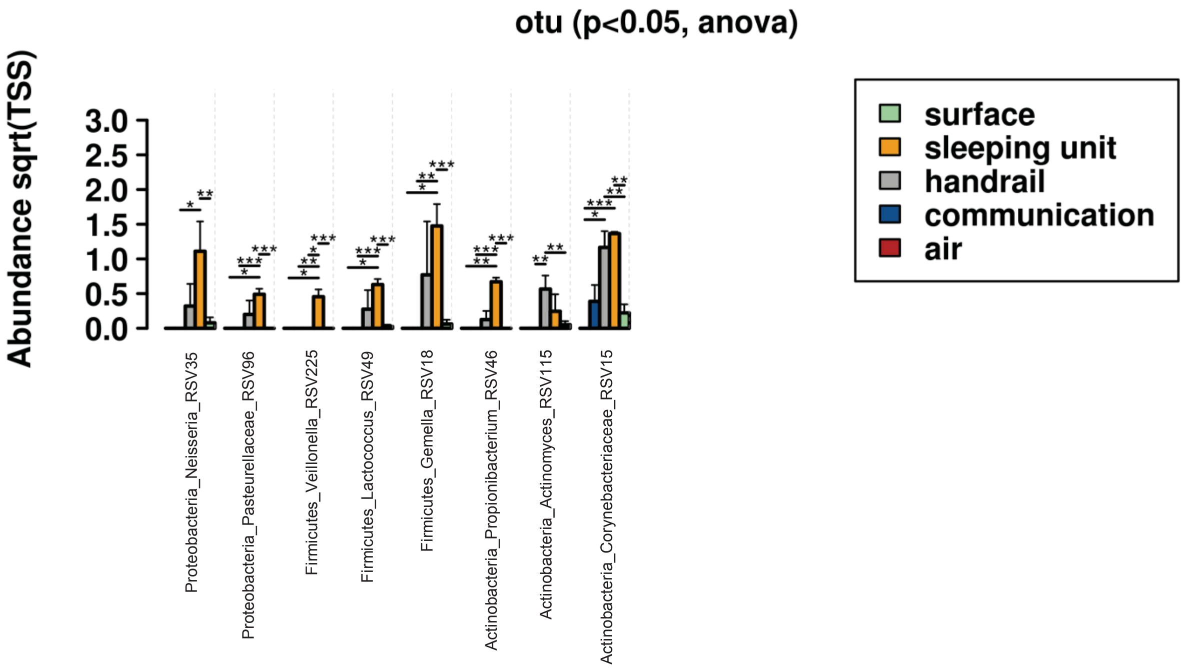

Supplementary Fig. 19 (refers to Supplementary Fig. 5, main manuscript): ANOVA plot analysis of different types of locations within the ISS and specifically associated RSVs. Error bars reflect standard deviation.

Supplementary Fig. 20

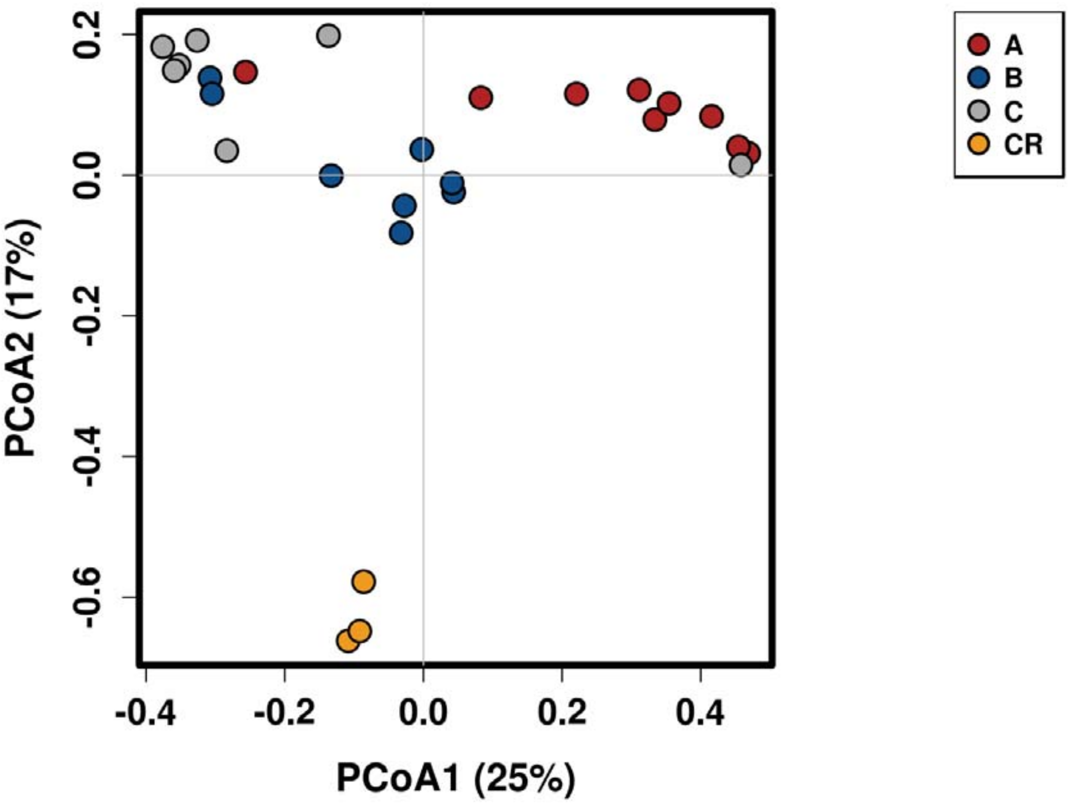

Supplementary Fig. 20 (refers to Supplementary Fig. 10, main manuscript): PCoA plot (based on Bray-Curtis) of microbiome compositions of clean room (CR) and ISS samples (Sessions A, B, C).

Supplementary Table 1

Higher taxonomy	Isolate	Origin	Biosafety risk group
**Ascomycota**			
**Dothideomycetes**	*Alternaria alternata*	CR	S1
	Aureobasidium pullulans	CR	S1
	Curvularia eragrostidis	CR	na
	Curvularia lunata	CR	S1
	Epicoccum sorghinum	CR	na
	Microsphaeropsis arundinis	CR	S1
	Phoma multirostrata	CR	na
**Eurotiomycetes**	*Aspergillus flavipes*	CR	S1
	Asperrgillus niger	CR	S2
	Aspergillus sydowii	A	S1
	Aspergillus unguis	A,CR	S1
	Penicillium aurantiogriseum	A, C	S1
	Penicillium brevicompactum	C	S1 allergenic
	Penicillium chermesinum	CR	na
	Penicillium chrysogenum	A, B, C	S1 allergenic
	Penicillium crustosum	A	S1 allergenic
	Penicillium expansum	A, C	S1
**Sordariomycetes**	*Chaetomium globosum*	A	S1
	Pestalotiopsis sp.	CR	na
	Trichoderma reesei	CR	S1
**Basidiomycota**			
**Microbotryomycetes**	*Rhodotorula mucilaginosa*	A,CR	S1 allergenic, opportunistic pathogen
**Zygomycota**			
**Zygomycetes**	*Rhizopus stolonifera*	A	S1 allergenic, black bread mold

Supplementary Table 1: List of fungal isolates in alphabetical order. Higher taxonomy is given in divisions and classes. Biosafety risk group information according to GESTIS (http://gestis.itrust.de). A= ISS Session A; B= ISS Session B; C= ISS Session C; CR =clean room; na= not listed in GESTIS.

Supplementary Table 2

Isolate	Upper limit NaCl (M)	Upper limit $MgSO_4.7H_2O$ (M)
Bacillus (para)licheniformis (pH5R25IB)	>2.0	1.4
Bacillus safensis (pH9R5IC)	>2.0	1.7
Paenibacillus campinasensis (pH9R2IIA)	1.8	1.5
Bacillus safensis (pH9R25IC)	>2.0	1.6
Paenibacillus glucanolyticus (R7C3IIIWC)	>2.0	1.8
Bacillus altitudinis (R10C4IIIB)	1.8	1.7
Roseomonas nepalensis (C55)	no growth	1.7
Bacillus nealsonii (pH7CW1HS0.2A)	1.3	1.6
Micrococcus yunnanensis (pH9R23IIA)	>2.0	>2.0
Micrococcus yunnanensis (pH9CD3IIA)	>2.0	>2.0
Staphylococcus arlettae (RAVCD2IIA)	>2.0	>2.0
Ralstonia insidiosa (RAVCW4IIA)	1.8	1.7
Staphylococcus capitis (R7AA8IB)	>2.0	1.8

Supplementary Table 2: Salt tolerance of selected isolates.
A subset of bacterial isolates was used to investigate salt tolerance against $MgSO_4x7H_2O$ and NaCl. Cells were inoculated into 7 ml TSB and incubated overnight in a shaking incubator (30°C, 100 rpm). Two molar stock solutions of NaCl and $MgSO_4x7H_2O$ were prepared in TSB media and filter sterilized (pore size: 0.22 µm). Serial solutions were then produced by mixing with salt-free media.
Optical density (600 nm) measurements were taken and an initial cell density of approximately 0.2±0.02 OD was used. Microorganisms were grown in 96-well plates in triplicates from concentrations of 0M up to 2M at increments of 0.1M, in both NaCl and $MgSO_4$. For each concentration, the wells contained 190 µl medium and 10 µl of the bacterial liquid culture. Plates were incubated for 48 hours at 28°C. Growth was defined as positive when OD readings were greater than 0.1. The highest concentration at which the cells grew was determined. Organisms displayed a tolerance of concentrations of $MgSO_4$ from 1.4 M to more than 2M $MgSO_4$ (*Micrococcus yunnanensis* and *Staphylococcus arlettae*) Higher concentrations could not be investigated on account of precipitation of salt in the chosen medium. Organisms displayed variable tolerance to NaCl.

Supplementary Table 3

Isolate ID	Closest neighbour	Similarity [%]	Sequence Accession No.
B8_ctrl_f_R2A	*Dyadobacter sediminis*	94.00	LR215112
B7_C100_a_R2A	*Glaciihabitans tibetensis*	97.19	LR215113
B2_ctrl_c_TSA	*Paenibacillus durus*	96.69	LR215117
HS_A2_IIA	*Paenibacillus tarimensis*	95.52	LR215085
B4_ctrl_b_R2A	*Paenibacillus tritici*	92.32	LR215118
R9_B3_IA	*Planococcus faecalis*	86.73	LR21510
B7_X125_a_R2A	*Spirosoma lacussanchae*	95.69	LR215121

Supplementary Table 3: List of putative novel isolates, as indicated based on partial 16S rRNA gene sequence comparison. The closest neighbour and similarity was determined using EzBioCloud[1].

Supplementary Table 4

Medium	Abb.	Target organisms	T [°C]	pH	Phase	Gas phase	Reference or manufacturer
R2A pH 4	R4	Acidophiles	32	4	Solid	Aerobic (ambient)	VWR Chemicals BDH Prolabo®
R2A pH 5	R5	Acidotolerants	32	5	Solid	Aerobic (ambient)	
R2A pH 7	R7W	Mesophiles	32	7	Solid	Aerobic (ambient)	
	R7K	Psychrophiles	4	7	Solid	Aerobic (ambient)	
	R7A	Anaerobes	32	7	Solid	N_2:H_2:CO_2 (80:10:10)	
R2A pH 9	R9	Alkalitolerants	32	9	Solid	Aerobic (ambient)	
R2A pH 10	R10	Alkaliphiles	32	10	Solid	Aerobic (ambient)	
RAVAN	RAV	Oligotrophs	32	7	Solid	Aerobic (ambient)	(2), (1:100 modified)
Halo medium	HLO	Halophiles	40	7.5	Solid	Aerobic (ambient)	DSMZ medium 97 (https://www.dsmz.de/)
Rogosa	ROG	Lactobacilli	32	5.5	Solid	Aerobic (ambient)	Merck
Tryptic Soy Agar	TSA	Mesophiles	32		Solid	Aerobic (ambient)	Becton Dickinson
Yeast-extract peptone medium	YPD	Yeasts & Fungi	32	7	Solid	Aerobic (ambient)	Sigma-Aldrich
Potato dextrose agar	PDA	Yeasts & Fungi	25	5.6	Solid	Aerobic (ambient)	Sigma-Aldrich
Autotrophic homoacetogen medium	AHM	Homoacetogen	32	7	Liquid	H_2:CO_2 (20:80)	(3)
Autotrophic allrounder medium	AAM	Autotrophs	32	7	Liquid	N_2:CO_2 (80:20)	(3)
Archaea supporting medium	ASM	Archaea	32	7	Liquid	H_2:CO_2 (20:80)	(3)
Medium for methanogens	MS	Methanogens	40	7	Liquid	H_2:CO_2 (20:80)	(4)
MS+organics	MSO	Methanogens	40	7	Liquid	H_2:CO_2 (20:80)	This study
N. exaquare medium	NEX	Thaumarchaea	32	8.5	Liquid	Aerobic (ambient)	(5)
Mixed ruminal bacteria medium	MCB-3	Methanogens	40	7	Liquid	H_2:CO_2 (20:80)	DSMZ medium322 (https://www.dsmz.de/)
R2A pH 7 liquid	R2A	Thermophiles	65	7	Liquid	Aerobic (ambient)	This study

Supplementary Table 4: Cultivation conditions and media.

Supplementary Table 5

Antibiotic substance	Type	Mechanism of action/target	Target group	Conc. applied (µg/ml)
Amoxicillin/ clavulanic acid	β- Lactam antibiotic (penicillin) and β-lactamase inhibitor	Inhibits cell wall synthesis; bactericidal	Gram + /-	0.016- 256
Ampicillin	β- Lactam antibiotic (penicillin)	Inhibits cell wall synthesis; bactericidal	Gram + /-	0.016- 256
Cefotaxime	β- Lactam antibiotic (cephalosporin)	Inhibits cell wall synthesis; bactericidal	Gram + /-	0.016– 32
Ceftriaxone	β- Lactam antibiotic; (cephalosporin)	Inhibits cell wall synthesis; bactericidal	Gram + /-	0.016– 256
Ciprofloxacin	Fluoroquinolone	Inhibits bacterial DNA gyrase; bactericidal	Gram + /-	0.002– 32
Clarithromycin	Macrolide	Inhibits protein synthesis; bacteriostatic	Gram + /-	0.016– 256
Clindamycin	Lincosamide	Inhibits protein synthesis; bacteriostatic	Gram + /-	0.016– 256
Colistin	Polypeptide antibiotic; polymyxin	Attacks cell membrane; bactericidal	Gram -	0.016– 256
Doxycycline	Polyketide antibiotic; (tetracycline)	Inhibits protein synthesis; bacteriostatic	Gram + /-	0.016– 256
Gentamicin	Aminoglycoside	Inhibits protein synthesis; bactericidal	Gram + /-	0.016– 256
Levofloxacin	Fluoroquinolone	Inhibits bacterial DNA gyrase; bactericidal	Gram + /-	0.002– 32
Linezolid	Oxazolidinone	Inhibits protein synthesis; bacteriostatic	Gram +	0.016– 256
Meropenem	β- Lactam antibiotic (carbapenem)	Inhibits cell wall synthesis; bactericidal	Gram + /-	0.002– 32
Moxifloxacin	Fluoroquinolone	Inhibits bacterial DNA gyrase; bactericidal	Gram + /-	0.002– 32
Penicillin G	β- Lactam antibiotic (penicillin)	Inhibits cell wall synthesis; bactericidal	Gram+	0.016– 256
Trimethoprim/sulfamethoxazole	Dihydrofolate reductase inhibtor and sulfonamide	Inhibits tetrahydrofolate synthesis; bactericidal	Gram + /-	0.002– 32
Vancomycin	Glycopeptide antibiotic	Inhibits cell wall synthesis; bactericidal	Gram+	0.016– 256

Supplementary Table 5: Antibiotics used for antimicrobial susceptibility tests, see also (6).

Supplementary Note 1

Supplementary Note 1: Genome analysis of selected bacterial isolates

The genome of *Bacillus pumilus* strain pH7_R2F_2_A was retrieved 99.59% complete, with a %GC of 41.6. The overall genome length was 3.7 Mbp. *Bacillus pumilus* SAFR-032 (3.7 Mbp, 41.3 %GC; ENA study ID: PRJNA20391), whose genome was analysed for comparative reasons as well, possessed the same antibiotics resistance capacity. The ISS strain possessed all necessary genes for flagellum assembly and CAS-TypeIIIB (with cmr5_TypeIIIB missing); the latter was not found in *Bacillus pumilus* SAFR-032. Looking at the metabolic profiles, the ISS isolate of *Bacillus pumilus* (comparison to SAFR-032 and ATCC 7061 (3.8 Mbp, 41.7 %GC; ENA study ID: PRJNA29785) possessed the genomic capacity to perform choline and methionine degradation, but no other peculiarities were identified.

The genome of *Bacillus safensis* strain pH9_R2_5_I_C was found to be 99.59% complete, with a %GC of 41.5. The overall genome length was 3.7 Mbp. It possessed all necessary genes for flagellum assembly and CAS-TypeIIIB, as did next neighbour *Bacillus safensis* FO-36b. Looking at the metabolic profiles, the ISS isolate of *Bacillus safensis* (comparison to CFA06 (3.7 Mbp, 41.5 %GC; ENA Study ID: PRJNA246604) and FO-36b (3.7 Mbp, 41.6 %GC; ENA Study ID: PRJNA270528) did not show certain peculiarities.

The genome of *Bradyrhizobium viridifuturi* strain pH5_R2_1_I_B was found to be 99.96% complete, with a %GC of 64.3. The overall genome length was 7.9 Mbp. The genome carried several copies of the efflux pump membrane transporter BepG as well as other multidrug efflux transporters and β-lactamase genes, which largely explained the overall stable antibiotic resistance observed in our experiments. The observed resistances against linezolid and vancomycin could not be directly inferred from the genomic data. These features were also found in *B. viridifuturi* SEMIA 690 (8.8 Mbp, 64.0 %GC; ENA Study ID: PRJNA290320), the next phylogenetic neighbour. Overall, the genetic features of our ISS isolate were widely similar to known *Bradyrhizobium* species. The only differences found were the potential capability for homospermidine biosynthesis from putrescine and (R)-acetoin biosynthesis through (S)-2-acetolactate. *B. viridifuturi* strain pH5_R2_1_I_B also possessed several heat shock proteins (hsp) which were not present in strain SEMIA 690 and explain how this isolate was able to survive the heat shock test applied in this study.

The genome of <u>*Cupriavidus metallidurans*</u> strain pH5_R2_1_II_A was found to be 99.94% complete, with a GC content of 63.7 %. The overall genome length was 6.9 Mbp. This strain carries three bepE efflux pump membrane transporters, and also a multidrug efflux system protein (acrB). However, the bepE efflux pumps were not detected in the genome of its closest relative *C. metallidurans* CH34. The genome showed full potential for type IV pili and flagella formation and numerous secretion systems, but this was not a unique feature for the ISS strain. With respect to the metabolic profile, *C.* <u>*metallidurans*</u> strain pH5_R2_1_II_A showed a number of different features when being compared to the next relatives (*C. basilensis* OR16, ENA Study ID: PRJNA79047; *C. metallidurans* CH34, ENA Study ID: PRJNA250; *C. necator* N-1, ENA Study ID: PRJNA67893; *C. taiwanensis* LMG19424, ENA Study ID: PRJNA15733), which included the predicted capacity for 5,6-dimethylbenzimidazole biosynthesis and butanediol degradation/synthesis.

The genome of <u>*Methylobacterium tardum*</u> strain pH5_R2_1_I_A was found to be 100% complete with a GC content of 69.2%, and a total genome length of 6.5 Mbp. Also *M. tardum* pH5_R2_1_I_A carried the efflux pump membrane transporter BepE and the genetic capacity for flagellum formation and several secretion systems. The strain showed a number of differential features when we compared the genomic potential with other members of the genus (*M. extorquens, M. mesophilicum, M. nodulans, M. populi, M. radiotolerans*; 5,6-dimethylbenzimidazole biosynthesis, base-degraded thiamine salvage, cytidylyl molybdenum cofactor biosynthesis, L-dopachrome biosynthesis); however, it shall be noted, that another genome of the species was not available at the time of analysis.

The genome of *Paenibacillus campinasensis* strain pH9_R2IIA could be retrieved with a 99.84% completeness. It showed a GC content of 52.26%, and a genome length of 5.4 Mbp. The genome revealed a potential for lincosamide (Clindamycin), macrolide (clarithromycin), fluorquinolone (moxifloxacin, levofloxacin, ciprofloxacin), and glycopeptide (vancomycin) resistance which could all be verified by the antimicrobial susceptibility tests with the exception of the vancomycin resistance (no PK/PD breakpoint in the EUCAST table). Nevertheless, the observed MIC for vancomycin was 4 µg/ml, which was the highest observed MIC for vancomycin besides the seven isolates which were completely resistant (see Fig. 6). The genome did not show any β-lactam resistances but in spite of this, *Paenibacillus campinasensis* strain pH9_R2IIA was resistant against all β-lactam antibiotics with the exception of meropenem in the antimicrobial susceptibility tests. The strain showed the potential for flagella formation, and the presence of CAS type III. At the time of the analysis there was no other genome of this species publically available, but the metabolic potential was not found to be strikingly different from other genome-sequenced members of the *Paenibacillus* genus.

Supplementary Note 2

Supplementary Note 2: Analysis of the decontam- dataset

In the main body of the manuscript we used a conservative approach and removed all RSVs detected in the negative controls from the dataset. In an alternative approach, we searched for contaminating RSVs also *via* „decontam“ [7]. Decontam identified 68 RSVs which were subsequently removed from the RSV table (identified contaminants, RSV tables before and after decontam purification shown in Supplementary Data 5).

However, the decontam RSV table still contained typical „kitome“ microbial signatures which were present in both, samples and negative controls ([8]; e.g. *Acinetobacter, Alcaligenes, Bacillus, Bradyrhizobium, Herbaspirillum, Mesorhizobium, Methylobacterium, Microbacterium, Novosphingobium, Pseudomonas, Ralstonia, Sphingomonas, Stenotrophomonas* and *Xanthomonas*). Based on this observation, we decided to use the conservative approach (all RSVs removed which were found in negative controls) for the main manuscript. For the sake of completeness, the analysis of the decontam- dataset is presented herein.

Supplementary References

1 Yoon, S.H., Ha, S.M., Kwon, S., Lim, J., Kim, Y., Seo, H. and Chun, J., 2017. Introducing EzBioCloud: a taxonomically united database of 16S rRNA gene sequences and whole-genome assemblies. *International journal of systematic and evolutionary microbiology*, *67*(5), p.1613.

2 Watve, M., Shejval, V., Sonawane, C., Rahalkar, M., Matapurkar, A., Shouche, Y., Patole, M., Phadnis, N., Champhenkar, A., Damle, K. and Karandikar, S., 2000. The'K'selected oligophilic bacteria: A key to uncultured diversity?. *Current science*, pp.1535-1542.

3 Stieglmeier, M., Wirth, R., Kminek, G. and Moissl-Eichinger, C., 2009. Cultivation of anaerobic and facultatively anaerobic bacteria from spacecraft-associated clean rooms. *Appl. Environ. Microbiol.*, *75*(11), pp.3484-3491.

4 Balch, W.E., Fox, G.E., Magrum, L.J., Woese, C.R. and Wolfe, R.S., 1979. Methanogens: reevaluation of a unique biological group. *Microbiological reviews*, *43*(2), p.260.

5 Sauder, L.A., Albertsen, M., Engel, K., Schwarz, J., Nielsen, P.H., Wagner, M. and Neufeld, J.D., 2017. Cultivation and characterization of Candidatus Nitrosocosmicus exaquare, an ammonia-oxidizing archaeon from a municipal wastewater treatment system. *The ISME journal*, *11*(5), p.1142.

6 Mora, M., Perras, A., Alekhova, T. A., Wink, L., Krause, R., Aleksandrova, A., Novozhilova, T. and Moissl-Eichinger, C. (2016) ‘Resilient microorganisms in dust samples of the International Space Station-survival of the adaptation specialists’, *Microbiome*, 4(1), p. 65. doi: 10.1186/s40168-016-0217-7.

7 Davis, N.M., Proctor, D.M., Holmes, S.P., Relman, D.A. and Callahan, B.J., 2018. Simple statistical identification and removal of contaminant sequences in marker-gene and metagenomics data. *Microbiome*, *6*(1), p.226.

8 Salter, S.J., Cox, M.J., Turek, E.M., Calus, S.T., Cookson, W.O., Moffatt, M.F., Turner, P., Parkhill, J., Loman, N.J. and Walker, A.W., 2014. Reagent and laboratory contamination can critically impact sequence-based microbiome analyses. *BMC biology*, *12*(1), p.87.
