# Supplementary material for: Space Station conditions are selective but do not alter microbial characteristics relevant to human health
Source: Nat Commun. 2019 Sep 5;10:3990. doi: 10.1038/s41467-019-11682-z (PMC6728350; doi:10.1038/s41467-019-11682-z)
Supplement: Supplementary file 3 — Description of Additional Supplementary Files [file 41467_2019_11682_MOESM3_ESM.pdf]

Description of Additional Supplementary Files

File Name: Supplementary Data 1
Description: RSV table of RSVs retrieved from ISS session A, B,C. Sample names refer to the wipe names (e.g. A-1, A-2 etc.) as given in Table 1.

File Name: Supplementary Data 2
Description: Taxonomic diversity as inferred from metagenomic dataset.

File Name: Supplementary Data 3
Description: Microbial functions obtained from shot-gun metagenomic sequencing.

File Name: Supplementary Data 4
Description: Unique isolates obtained from ISS samples and partial 16S rRNA gene (Sanger sequencing).

File Name: Supplementary Data 5
Description: Decontam dataset, including the RSV table before decontam processing, RSV table after decontam purification and identified contaminants.
