# Supplementary material for: Space Station conditions are selective but do not alter microbial characteristics relevant to human health
Source: Nat Commun. 2019 Sep 5;10:3990. doi: 10.1038/s41467-019-11682-z (PMC6728350; doi:10.1038/s41467-019-11682-z)
Supplement: Supplementary file 9 — Reporting Summary [file 41467_2019_11682_MOESM9_ESM.pdf]

nature research

Corresponding author(s): Christine Moissl-Eichinger

Last updated by author(s): 2019_07_15

Reporting Summary

Nature Research wishes to improve the reproducibility of the work that we publish. This form provides structure for consistency and transparency in reporting. For further information on Nature Research policies, see Authors & Referees and the Editorial Policy Checklist.

Statistics

For all statistical analyses, confirm that the following items are present in the figure legend, table legend, main text, or Methods section.

n/a	Confirmed	
☐	☒	The exact sample size (n) for each experimental group/condition, given as a discrete number and unit of measurement
☒	☐	A statement on whether measurements were taken from distinct samples or whether the same sample was measured repeatedly
☐	☒	The statistical test(s) used AND whether they are one- or two-sided<br>*Only common tests should be described solely by name; describe more complex techniques in the Methods section.*
☒	☐	A description of all covariates tested
☐	☒	A description of any assumptions or corrections, such as tests of normality and adjustment for multiple comparisons
☒	☐	A full description of the statistical parameters including central tendency (e.g. means) or other basic estimates (e.g. regression coefficient) AND variation (e.g. standard deviation) or associated estimates of uncertainty (e.g. confidence intervals)
☒	☐	For null hypothesis testing, the test statistic (e.g. F, t, r) with confidence intervals, effect sizes, degrees of freedom and P value noted<br>*Give P values as exact values whenever suitable.*
☒	☐	For Bayesian analysis, information on the choice of priors and Markov chain Monte Carlo settings
☒	☐	For hierarchical and complex designs, identification of the appropriate level for tests and full reporting of outcomes
☒	☐	Estimates of effect sizes (e.g. Cohen's d, Pearson's r), indicating how they were calculated

Our web collection on statistics for biologists contains articles on many of the points above.

Software and code

Policy information about availability of computer code

Data collection	see comment below. all stated in Materials and methods.
Data analysis	Demultiplexed, paired reads were processed in R (version 3.2.2) using the R package DADA2 as described in 67. In brief, sequences were quality checked, filtered, and trimmed to a consistent length of ~270 bp (universal primer set) and ~140 bp (archaeal primer set). The trimming and filtering were performed on paired end reads with a maximum of two expected errors per read (maxEE = 2). Passed sequences were de-replicated and subjected to the DADA2 algorithm to identify indel-mutations and substitutions. The DADA2 output table is not based on a clustering step and thus no operational taxonomic units (OTUs) were generated. Each row in the DADA2 output table corresponds to a non-chimeric inferred sample sequence (ribosomal sequence variants; RSVs) 67. In addition, the merging step occurs after denoising, which increases accuracy. After merging paired end reads and chimera filtering, taxonomy was assigned with the RDP classifier and the SILVA v.123 trainset 68. The visualization was carried out using the online software suite Calypso 69. For bar plots data was normalized by total sum normalization (TSS) and for PCoA/NMDS by TSS combined with square root transformation. For the Shannon index analysis, sequences were rarefied as indicated in the Figure captions. Tax4fun was performed based on the Silva-classified OTU table, as described 70.<br>Microbial Community Network. G-test for independence and edge weights were calculated on the RSV table using the make_otu_network.py script in QIIME 1.9.1 71. The network table with calculated statistics was then imported into Cytoscape 3.7.1 72 and visualized as a bipartite network of sample (hexagons) and RSV nodes (circles) connected by edges. For clustering, a stochastic spring-embedded algorithm based on the calculated edge weights was used. Size, transparency and labels were correlated with RSV abundances, border line intensity refers to RSV persistence over multiple sampling sessions and edge transparency was correlated to calculated edge weights.<br>Shotgun metagenomics. Shotgun libraries for Illumina MiSeq sequencing were prepared with the NEBNext® Ultra II DNA Library Prep Kit for Illumina® in combination with the Index Primer Set 1 (NEB, Frankfurt, Germany) according to manufacturer's instructions and as described in 73. Briefly, 500 ng of dsDNA were randomly fragmented by ultrasonication in a microTUBE on a M220 Focused-ultrasonicator™ (Covaris, USA) in a total volume of 130 µl 1xTE for 80 seconds with 200 cycles per burst (140 peak incident power, 10% duty factor). After shearing, 200 ng of sheared DNA were used for the end repair and adapter ligation reactions in the NEBNext® Ultra II DNA Library Prep Kit for Illumina® according to manufacturer's instructions. Size selection and purification were performed according to

the instructions for 300 to 400bp insert size. Subsequent PCR amplification was performed with 4 cycles and libraries were eluted after successful amplification and purification in 33 µl 1xTE buffer pH 8.0. For quality control libraries were analyzed with a DNA High Sensitivity Kit on a 2100 Bioanalyzer system (Agilent Technologies, USA) and again quantified on a Quantus™ Fluorometer (Promega, Germany). An equimolar pool was sequenced on an Illumina MiSeq desktop sequencer (Illumina, CA, USA). Libraries were diluted to 8 pM and run with 5% PhiX and v3 600 cycles chemistry according to manufacturer's instructions. Raw fastq data files were uploaded to the metagenomics analysis server (MG-RAST) 74 and processed with default parameters. Annotations of taxonomy (RefSeq) and functions (Subsystems) were then imported to QIIME 2 (2018.11) 75 or Calypso 69 to calculate core features, alpha and beta diversity metrics, statistics and additional visualizations of the datasets. Additional statistical analyses were performed using STAMP 76

For manuscripts utilizing custom algorithms or software that are central to the research but not yet described in published literature, software must be made available to editors/reviewers. We strongly encourage code deposition in a community repository (e.g. GitHub). See the Nature Research guidelines for submitting code & software for further information.

Data

Policy information about availability of data

All manuscripts must include a data availability statement. This statement should provide the following information, where applicable:

- Accession codes, unique identifiers, or web links for publicly available datasets
- A list of figures that have associated raw data
- A description of any restrictions on data availability

The assembled genomes are available through MaGe and EMBL/ENA (accession number PRJEB30995). Raw 16S rRNA gene sequences of the molecular approach are available in the European nucleotide archive, study ID: PRJEB30994. The partial 16S rRNA gene sequences of unique bacterial isolates obtained during the EXTREMOPHILES flight project and from the S5C cleanroom in Kourou are available via ENA study ID PRJEB30998 and Supplementary Data 2.

Field-specific reporting

Please select the one below that is the best fit for your research. If you are not sure, read the appropriate sections before making your selection.

☒ Life sciences ☐ Behavioural & social sciences ☐ Ecological, evolutionary & environmental sciences

For a reference copy of the document with all sections, see nature.com/documents/nr-reporting-summary-flat.pdf

Life sciences study design

All studies must disclose on these points even when the disclosure is negative.

Sample size	Sample size was limited by the experiment design (sampling aboard the International space station). The limitations of the sampling are discussed in the manuscript.
Data exclusions	N.a.
Replication	N.a.
Randomization	N.a.
Blinding	N.a.

Reporting for specific materials, systems and methods

We require information from authors about some types of materials, experimental systems and methods used in many studies. Here, indicate whether each material, system or method listed is relevant to your study. If you are not sure if a list item applies to your research, read the appropriate section before selecting a response.

Materials & experimental systems

n/a	Involved in the study	
☒	☐	Antibodies
☒	☐	Eukaryotic cell lines
☒	☐	Palaeontology
☒	☐	Animals and other organisms
☒	☐	Human research participants
☒	☐	Clinical data

Methods

n/a	Involved in the study	
☒	☐	ChIP-seq
☒	☐	Flow cytometry
☒	☐	MRI-based neuroimaging
