# Supplementary material for: Space Station conditions are selective but do not alter microbial characteristics relevant to human health
Source: Nat Commun. 2019 Sep 5;10:3990. doi: 10.1038/s41467-019-11682-z (PMC6728350; doi:10.1038/s41467-019-11682-z)
Supplement: Supplementary file 10 — Source Data [file 41467_2019_11682_MOESM10_ESM.pptx]

## Slide 1
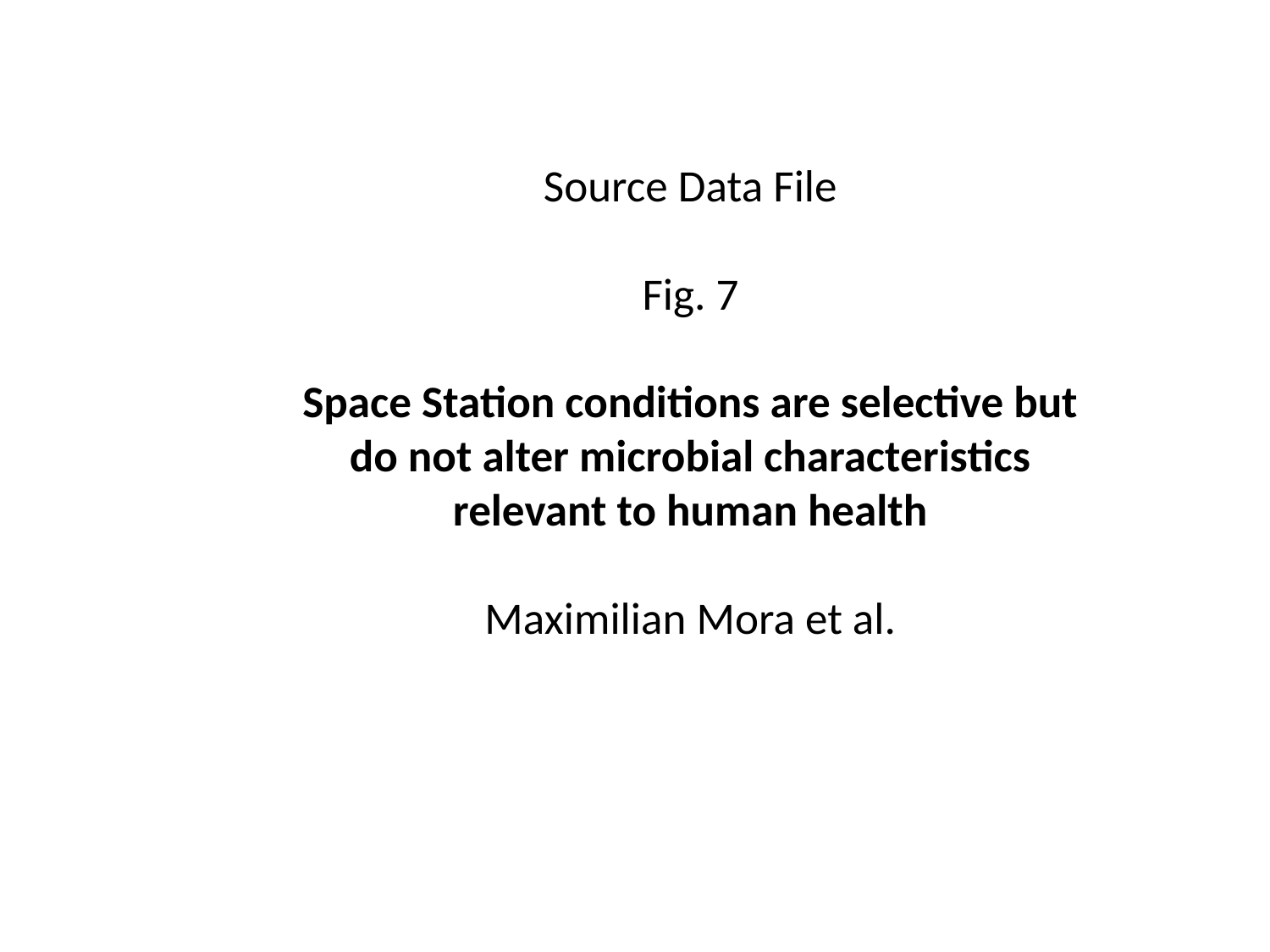

Source Data File
Fig. 7
Space Station conditions are selective but do not alter microbial characteristics relevant to human health
 
Maximilian Mora et al.

## Slide 2
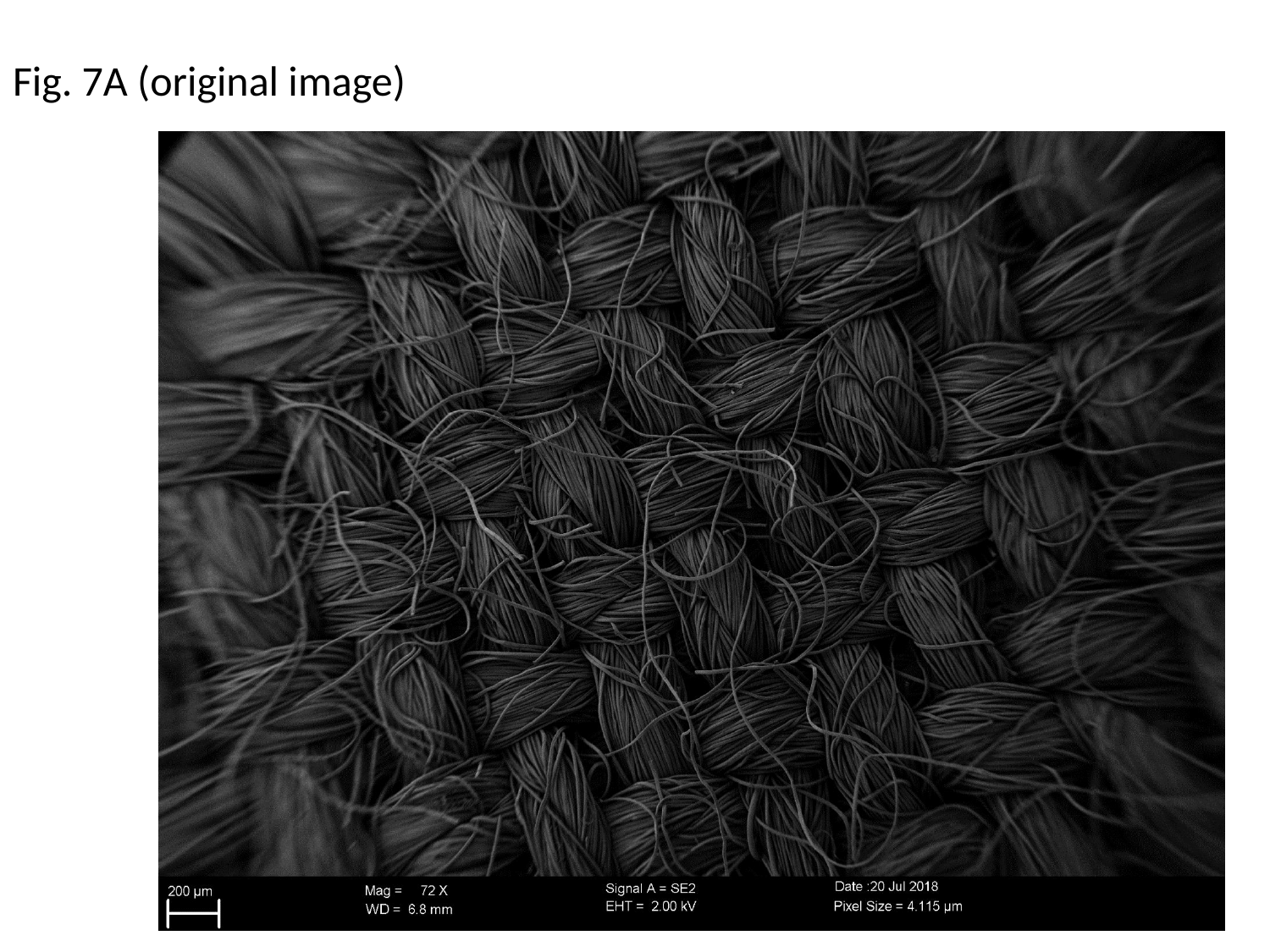

# Fig. 7A (original image)

## Slide 3
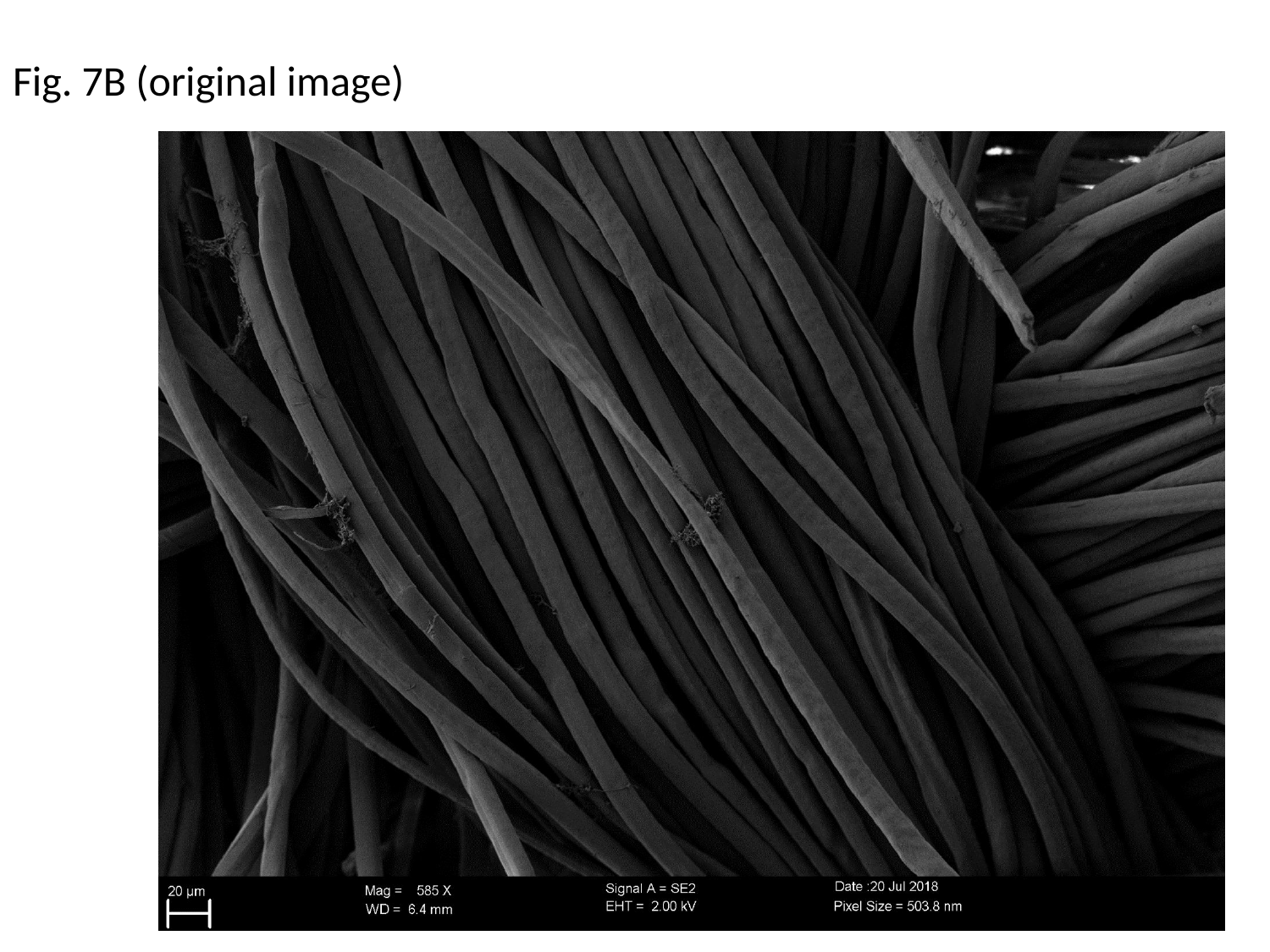

# Fig. 7B (original image)

## Slide 4
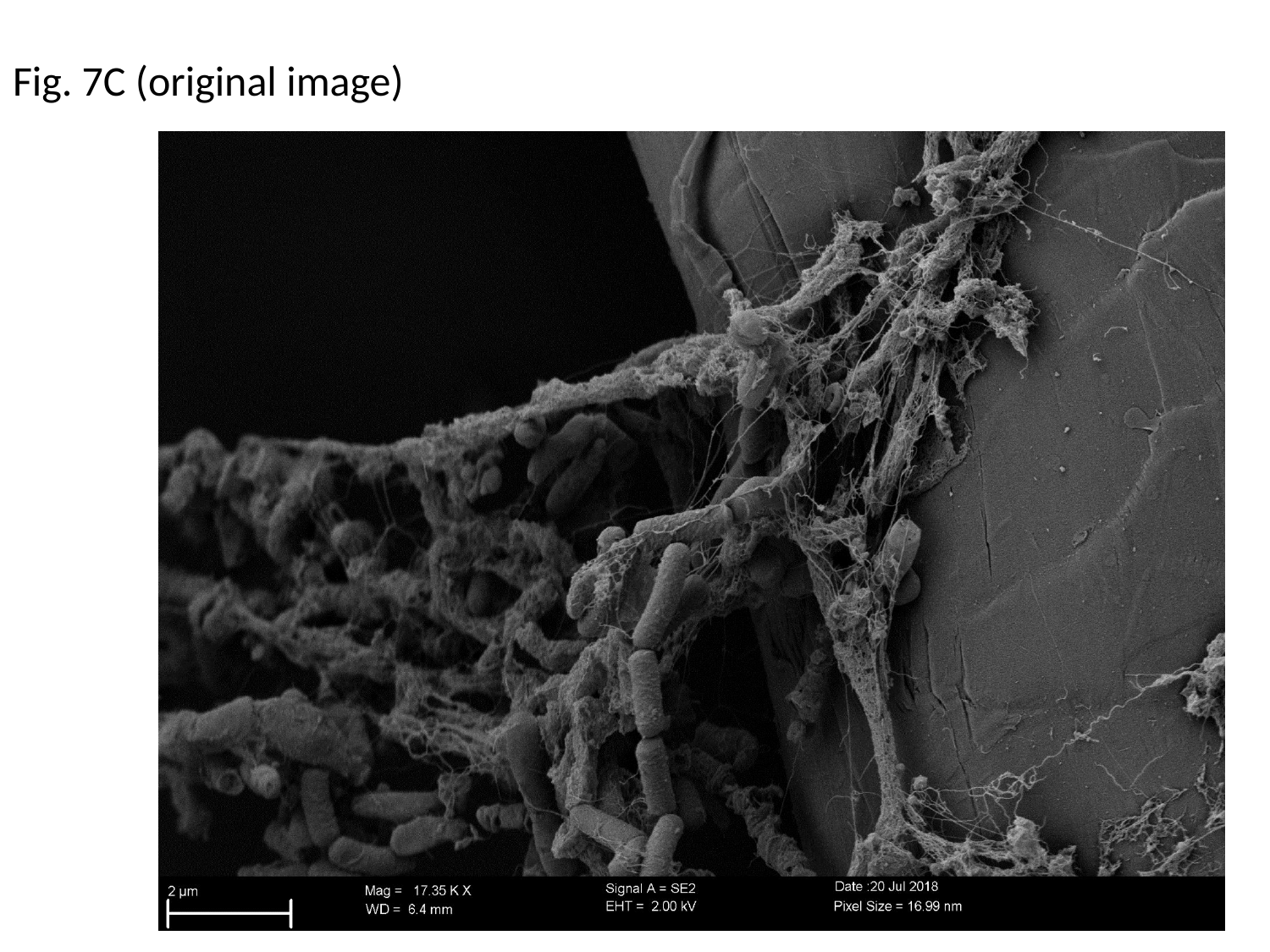

# Fig. 7C (original image)

## Slide 5
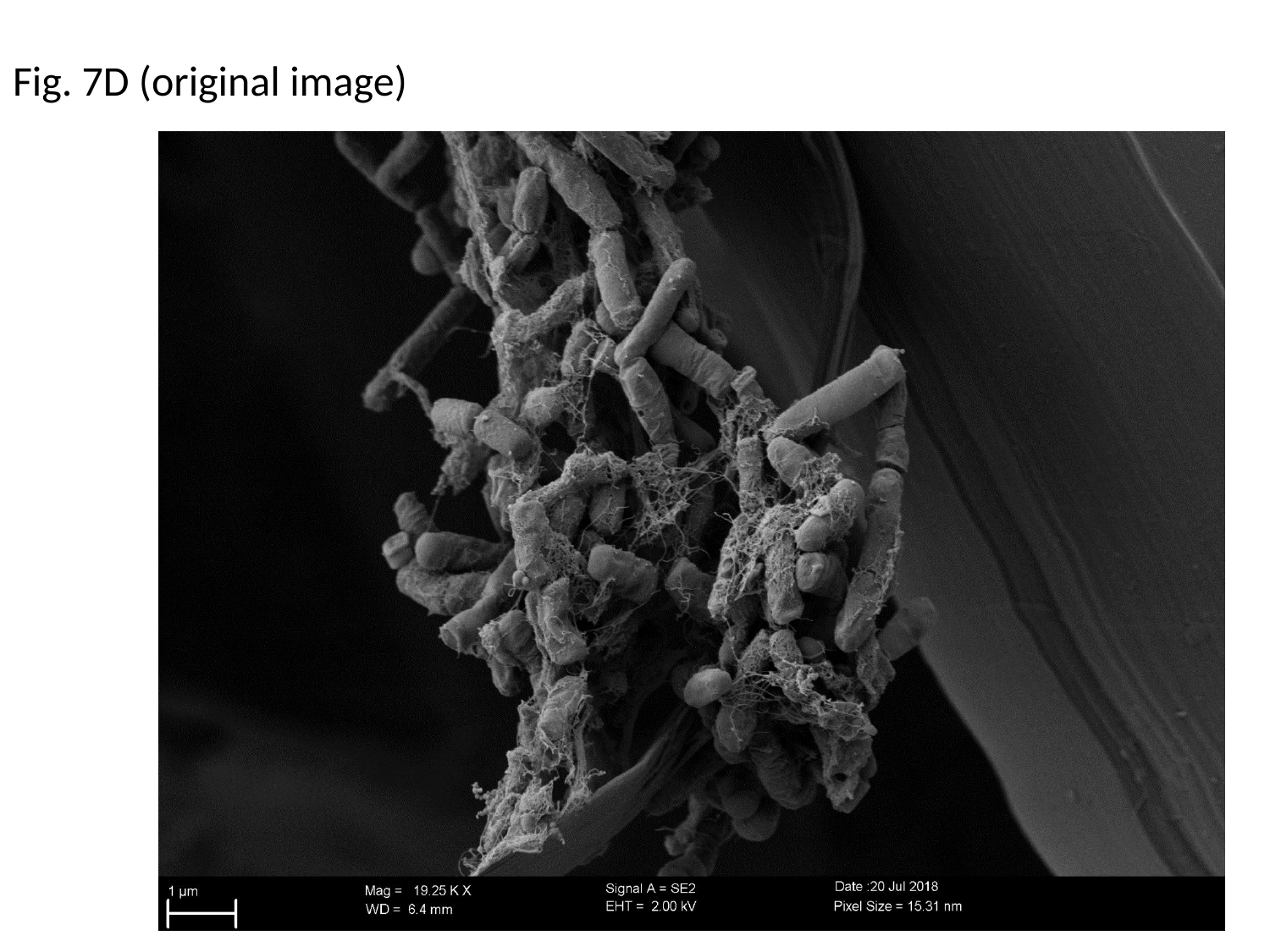

# Fig. 7D (original image)

## Slide 6
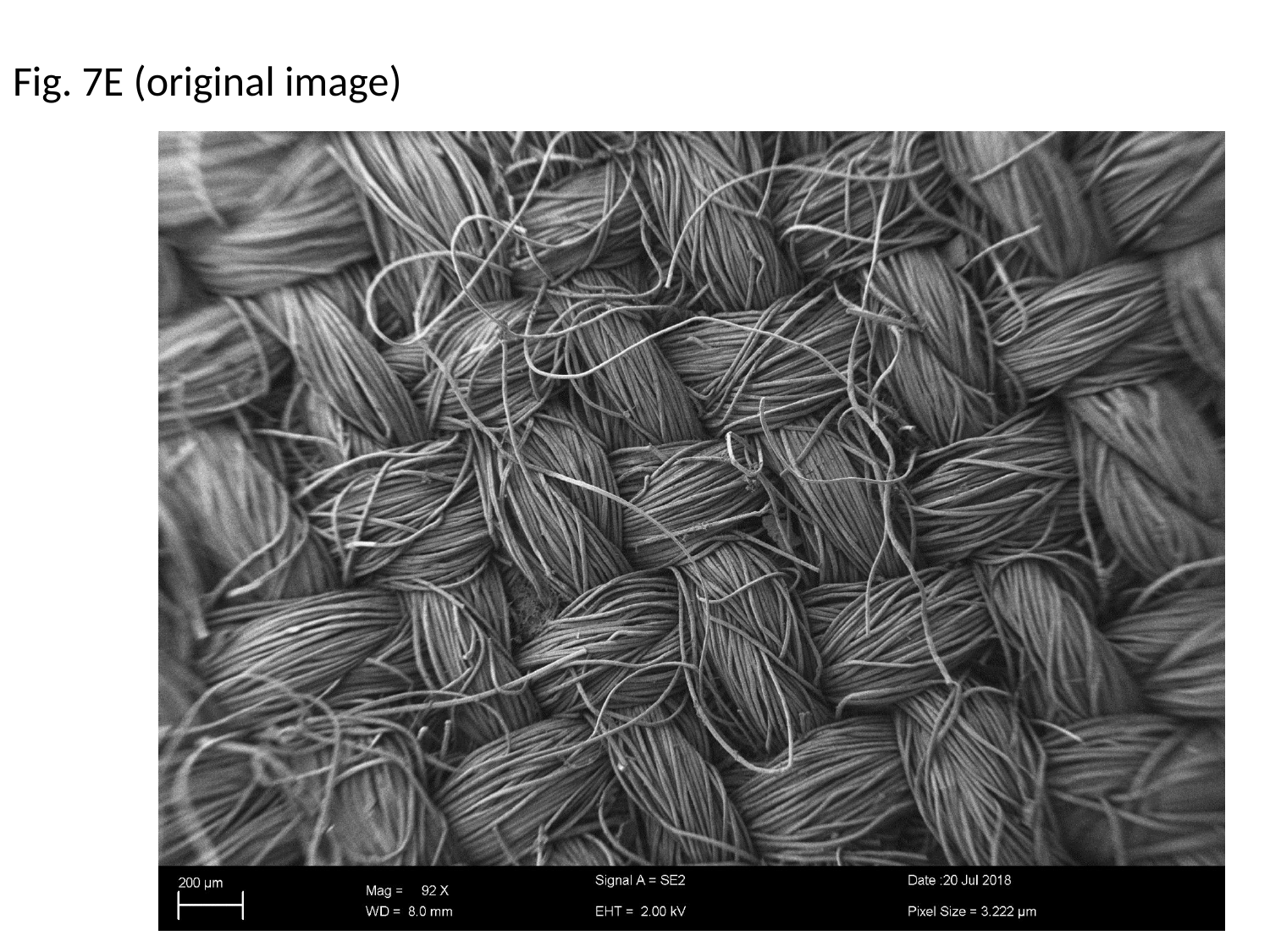

# Fig. 7E (original image)

## Slide 7
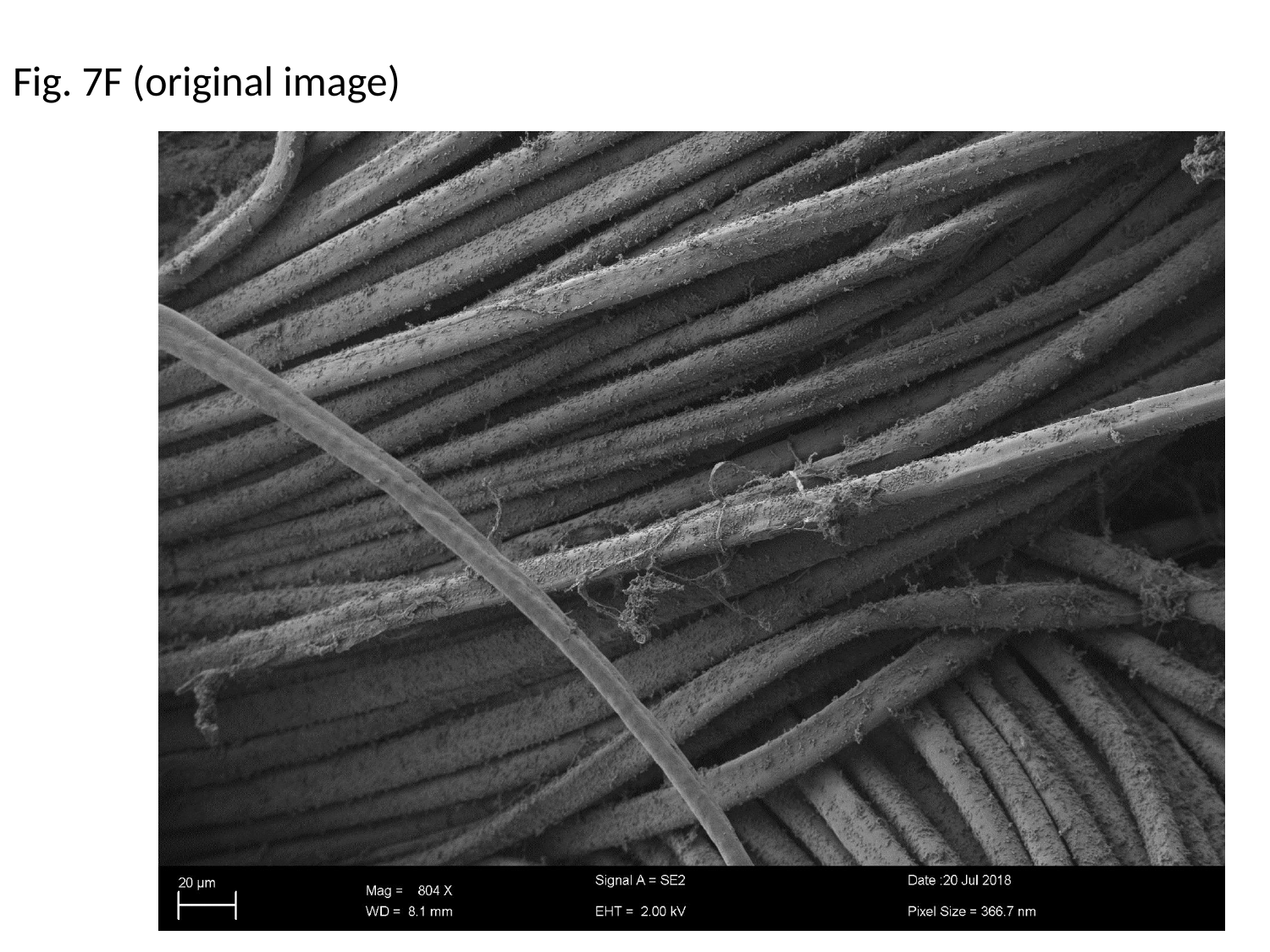

# Fig. 7F (original image)

## Slide 8
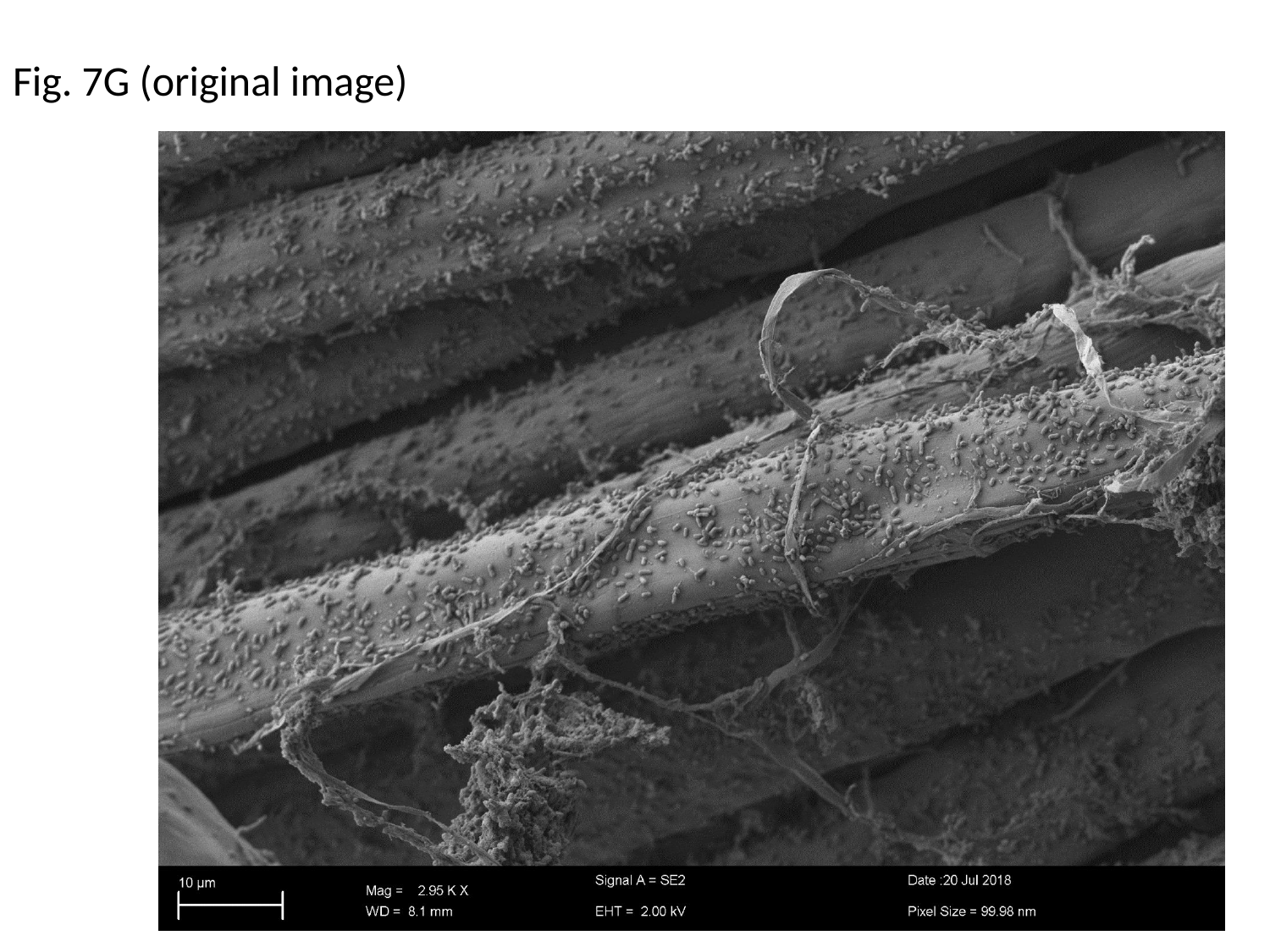

# Fig. 7G (original image)

## Slide 9
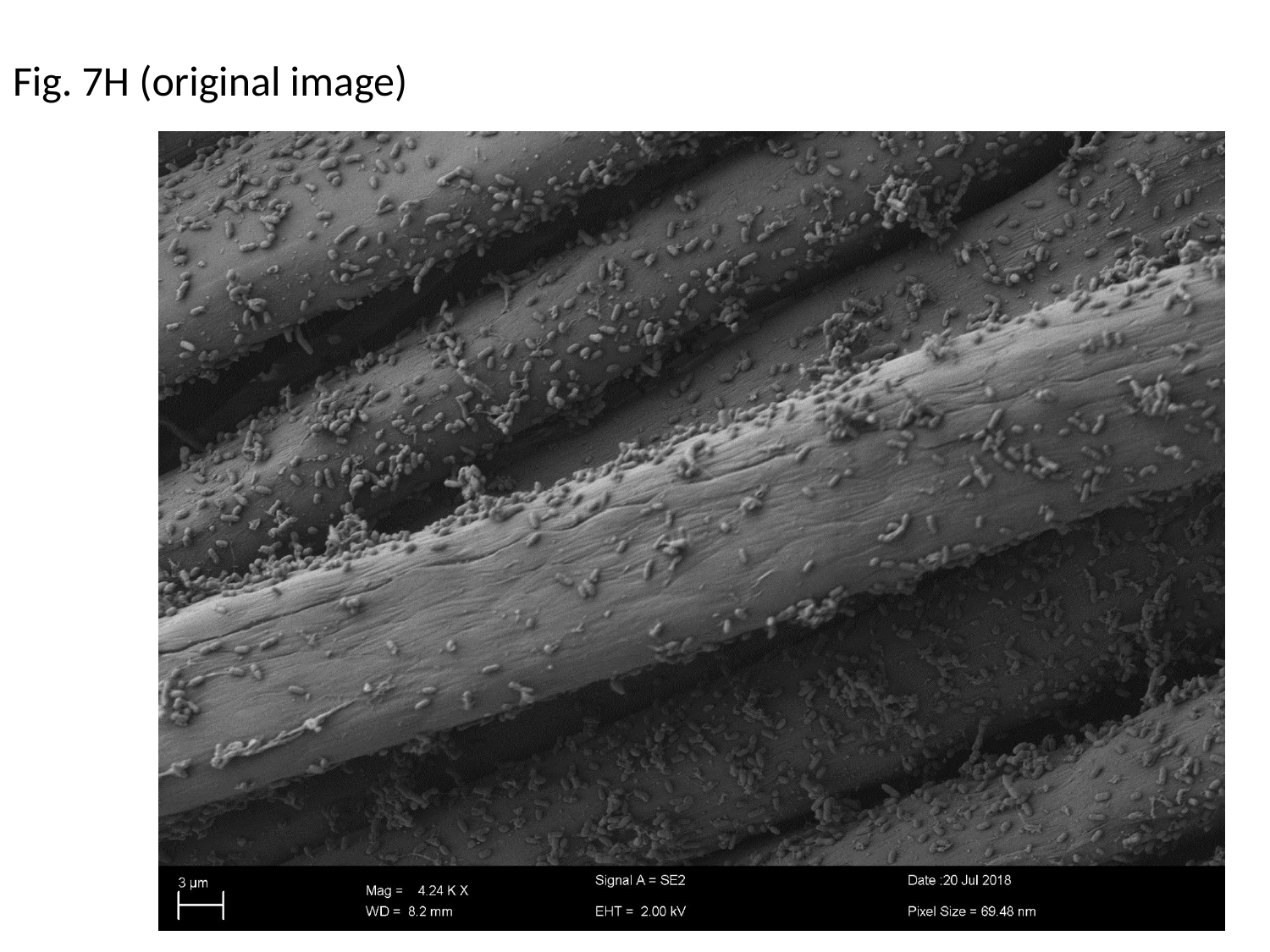

# Fig. 7H (original image)

## Slide 10
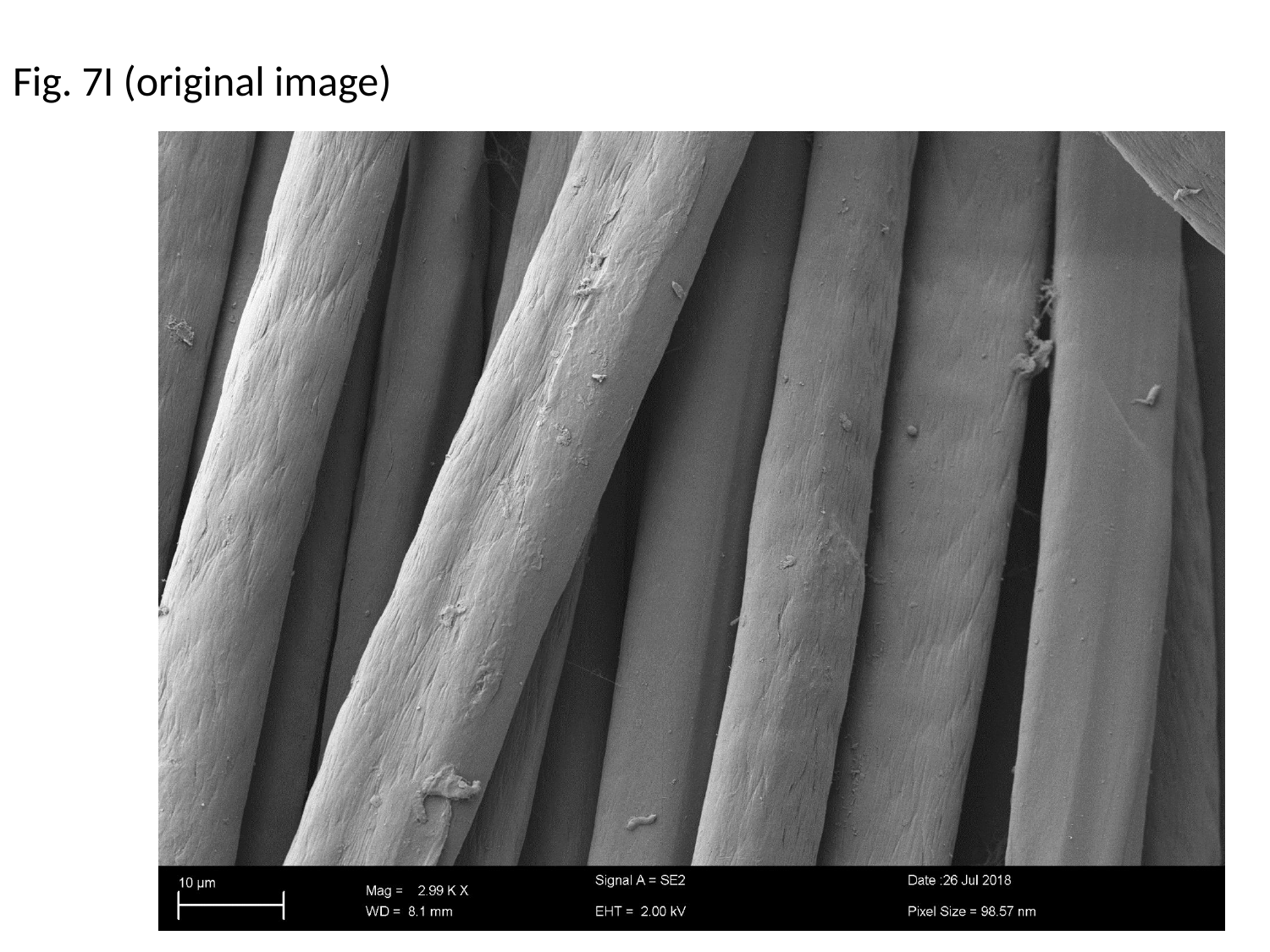

# Fig. 7I (original image)

## Slide 11
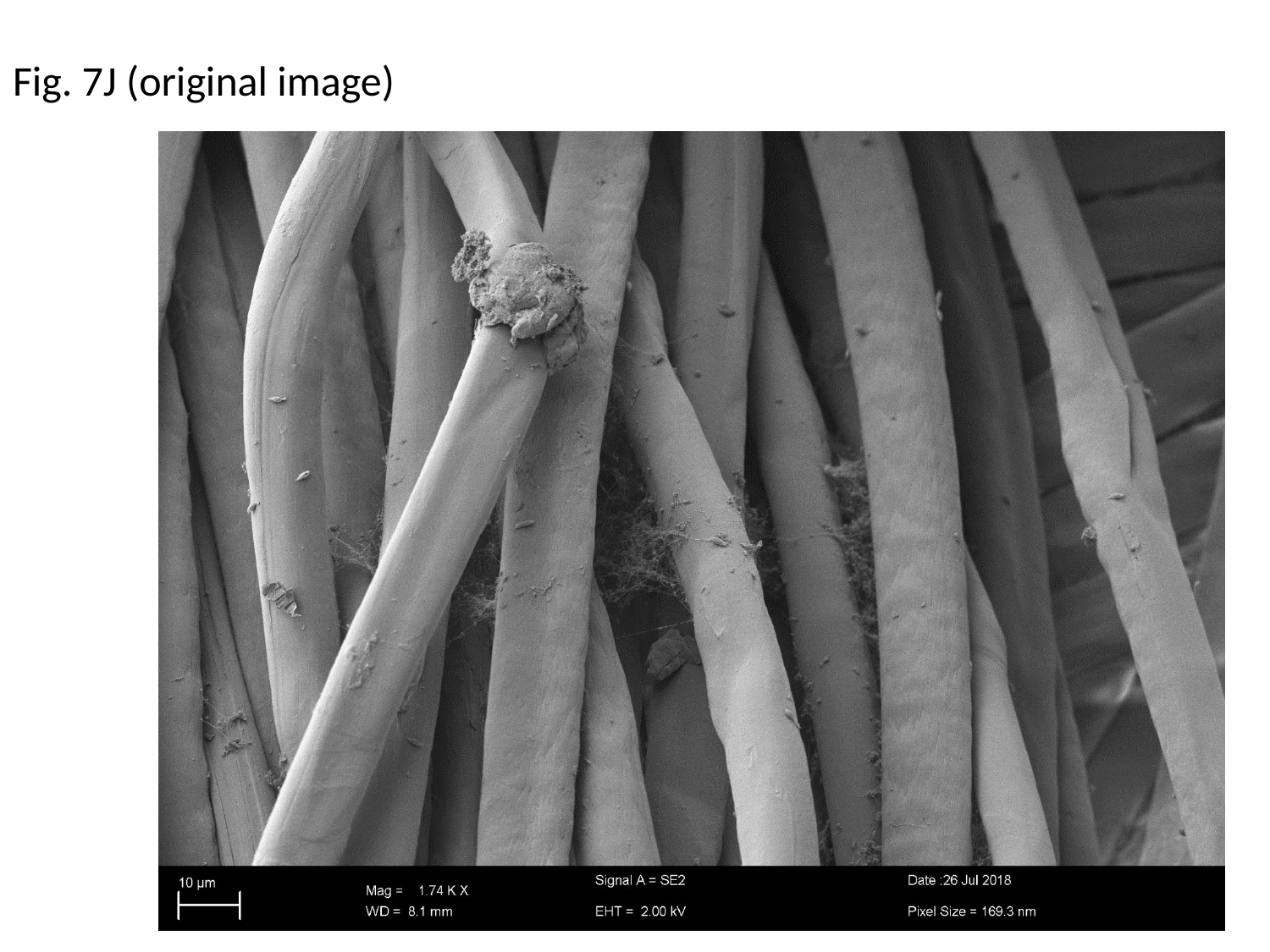

# Fig. 7J (original image)

## Slide 12
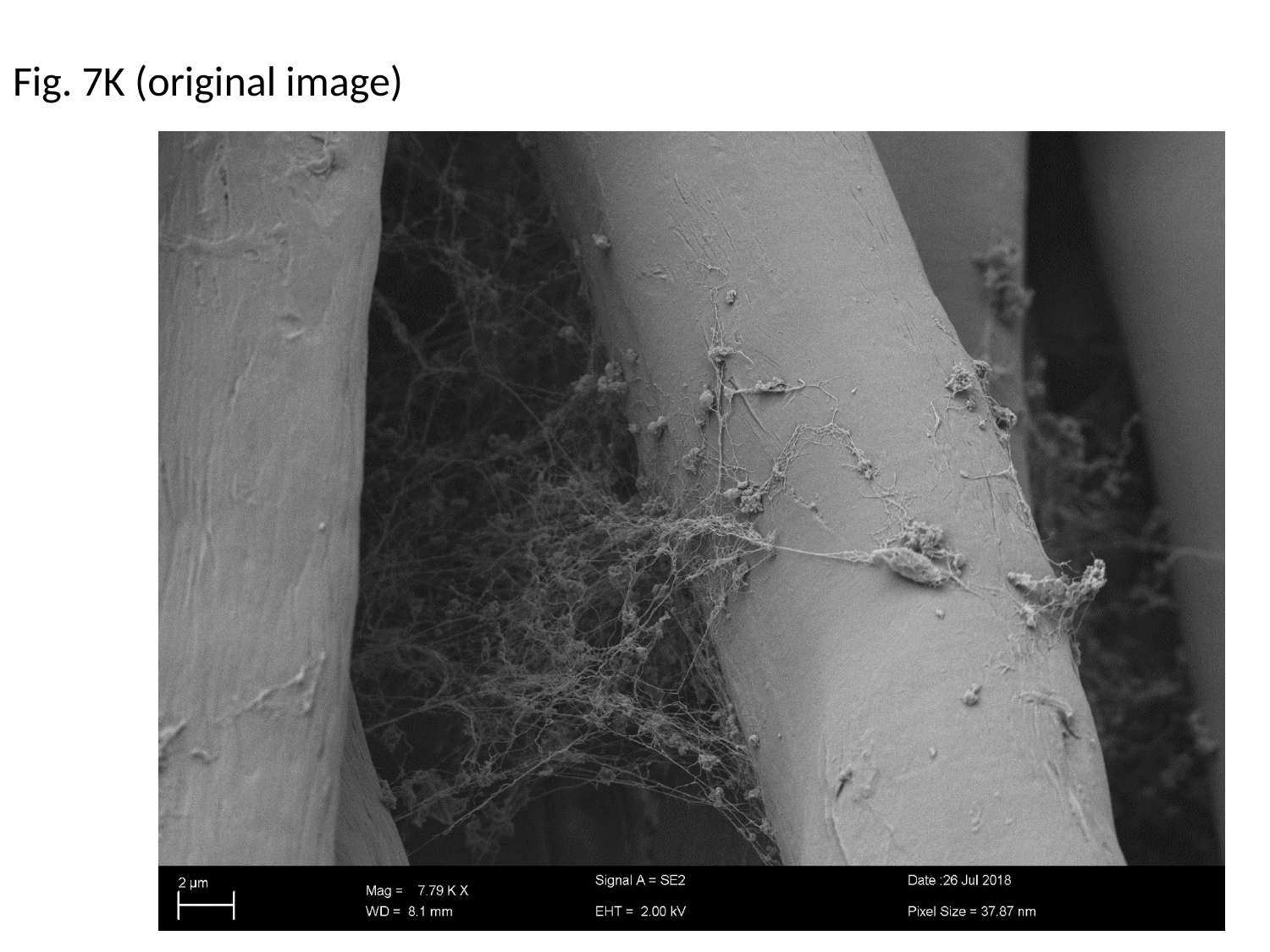

# Fig. 7K (original image)

## Slide 13
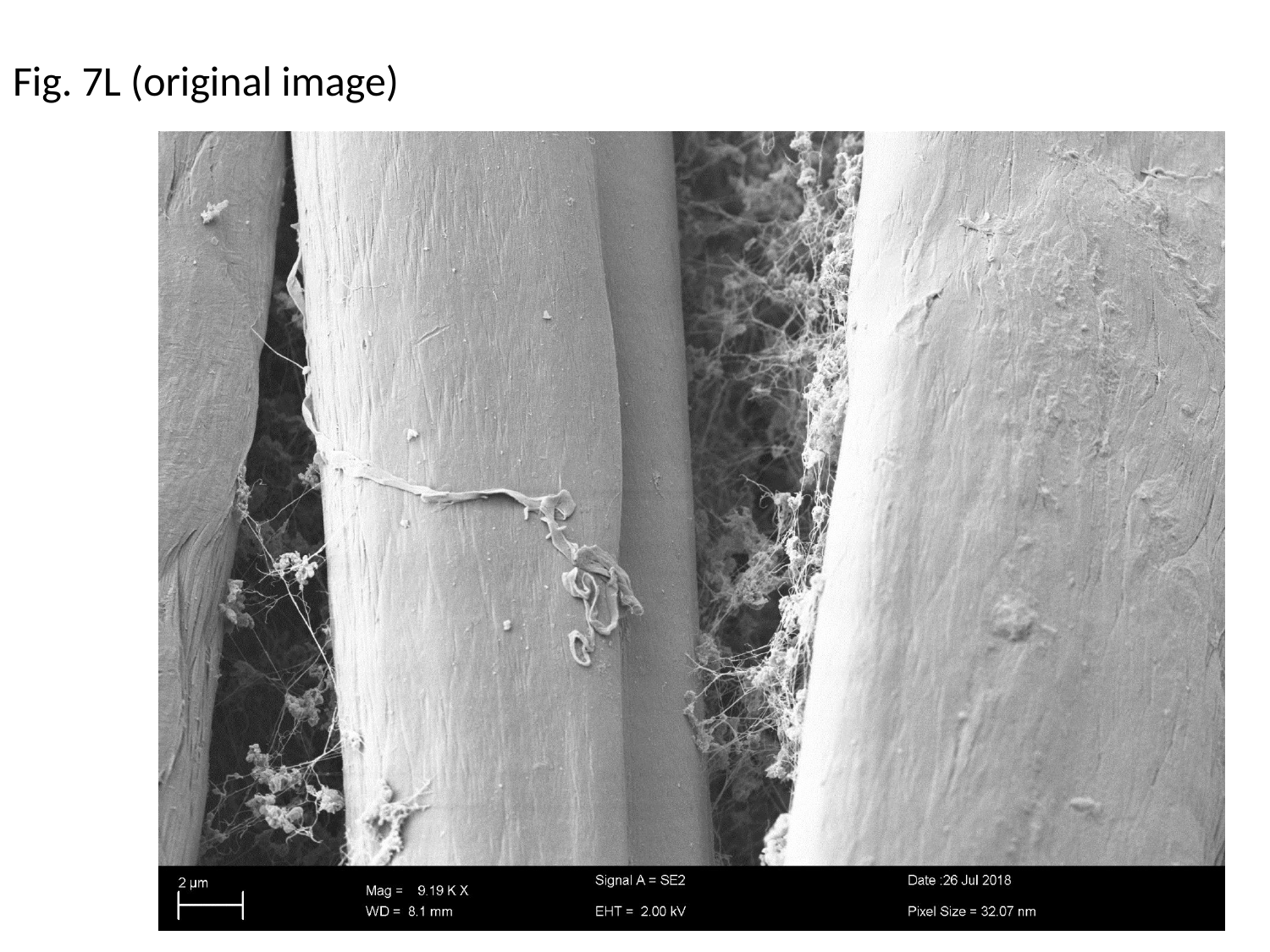

# Fig. 7L (original image)
